# Elevated plasma succinate levels are linked to higher cardiovascular disease risk factors in young adults

**DOI:** 10.1186/s12933-021-01333-3

**Published:** 2021-07-27

**Authors:** Francisco J. Osuna-Prieto, Borja Martinez-Tellez, Lourdes Ortiz-Alvarez, Xinyu Di, Lucas Jurado-Fasoli, Huiwen Xu, Victoria Ceperuelo-Mallafré, Catalina Núñez-Roa, Isabelle Kohler, Antonio Segura-Carretero, José V. García-Lario, Angel Gil, Concepción M. Aguilera, Jose M. Llamas-Elvira, Patrick C. N. Rensen, Joan Vendrell, Jonatan R. Ruiz, Sonia Fernández-Veledo

**Affiliations:** 1 PROFITH (PROmoting FITness and Health Through Physical Activity) Research Group, Department of Physical Education and Sport, Faculty of Sport Sciences, University of Granada, Granada, Spain; 2 Department of Analytical Chemistry, University of Granada, Granada, Spain; 3 Research and Development of Functional Food Center (CIDAF), Granada, Spain; 4 Division of Endocrinology and Einthoven Laboratory for Experimental Vascular Medicine, Department of Medicine, Leiden University Medical Center, Leiden, The Netherlands; 5 Division of Systems Biomedicine and Pharmacology, Leiden Academic Center for Drug Research, Leiden University, Leiden, The Netherlands; 6 Department of Endocrinology and Nutrition and Research Unit, University Hospital of Tarragona Joan XXIII-Institut d ´Investigació Sanitària Pere Virgili (IISPV), Tarragona, Spain; 7 CIBER de Diabetes y Enfermedades Metabólicas Asociadas (CIBERDEM)-Instituto de Salud Carlos III, Madrid, Spain; 8 Rovira i Virgili University, Tarragona, Spain; 9 Division of BioAnalytical Chemistry, Vrije Universiteit Amsterdam, Amsterdam Institute of Molecular and Life Sciences (AIMMS), Amsterdam, The Netherlands; 10 Center for Analytical Sciences Amsterdam, Amsterdam, The Netherlands; 11 Hospital Universitario Clínico San Cecilio, Granada, Spain; 12 Department of Biochemistry and Molecular Biology II, “José Mataix Verdú” Institute of Nutrition and Food Technology (INYTA), Biomedical Research Center (CIBM), University of Granada, Granada, Spain; 13 Biohealth Research Institute in Granada (Ibs, GRANADA), Granada, Spain; 14 CIBER Fisiopatología de la Obesidad y la Nutrición (CIBEROBN), Madrid, Spain; 15 Nuclear Medicine Service, Virgen de las Nieves University Hospital, Biohealth Research Institute in Granada (Ibs. GRANADA), Granada, Spain

**Keywords:** Obesity, Visceral adiposity, Inflammation, Oxylipins, Succinate

## Abstract

**Background:**

Succinate is produced by both host and microbiota, with a key role in the interplay of immunity and metabolism and an emerging role as a biomarker for inflammatory and metabolic disorders in middle-aged adults. The relationship between plasma succinate levels and cardiovascular disease (CVD) risk in young adults is unknown.

**Methods:**

Cross-sectional study in 100 (65% women) individuals aged 18–25 years from the ACTIvating Brown Adipose Tissue through Exercise (ACTIBATE) study cohort. CVD risk factors, body composition, dietary intake, basal metabolic rate, and cardiorespiratory fitness were assessed by routine methods. Plasma succinate was measured with an enzyme-based assay. Brown adipose tissue (BAT) was evaluated by positron emission tomography, and circulating oxylipins were assessed by targeted metabolomics. Fecal microbiota composition was analyzed in a sub-sample.

**Results:**

Individuals with higher succinate levels had higher levels of visceral adipose tissue (VAT) mass (+ 42.5%), triglycerides (+ 63.9%), C-reactive protein (+ 124.2%), diastolic blood pressure (+ 5.5%), and pro-inflammatory omega-6 oxylipins than individuals with lower succinate levels. Succinate levels were also higher in metabolically unhealthy individuals than in healthy overweight/obese peers. Succinate levels were not associated with BAT volume or activity or with fecal microbiota composition and diversity.

**Conclusions:**

Plasma succinate levels are linked to a specific pro-inflammatory omega-6 signature pattern and higher VAT levels, and seem to reflect the cardiovascular status of young adults.

**Supplementary Information:**

The online version contains supplementary material available at 10.1186/s12933-021-01333-3.

## Background

Cardiovascular disease (CVD) remains the main cause of death worldwide [1]. Worryingly, the rates of CVD are increasing in young/middle-aged adults (18–45 years) [2]. The incorporation of new circulating biochemical markers and technologies are improving the detection of CVD risk in the general population [3, 4]. Early identification of individuals at risk of developing CVD is important [5], but predictive biomarkers of CVD risk and related metabolic disturbances are not well characterized in young adults [6]. Advances in the functional analysis of the human metabolome have yielded many new endogenous metabolites as potential biomarkers for CVD [7], including the tricarboxylic acid (TCA) cycle intermediate succinate [8].

Historically considered as a respiratory substrate of the mitochondrial electron transport chain, succinate is now known to have additional physiological roles. For example, it acts as a signaling molecule in both intracellular and extracellular compartments by binding and activating its cognate receptor, succinate receptor 1 (SUCNR1), also known as G-protein coupled receptor 91 [9]. In addition to being a marker of hypoxia and a driver of tissue damage [10], succinate is now recognized as a pro-inflammatory signal that boosts immune activation [11–13]. We and others have shown that succinate also plays a key role in the fine-tuning of the inflammatory response, acting both as an alarmin [11–13] and as a resolving molecule [14–17]. Moreover, succinate is a positive regulator of intestinal gluconeogenesis [18], activates brown adipose tissue (BAT) thermogenesis [19], and is involved in the muscle-remodeling program in response to exercise [20, 21]. Additional roles for succinate in energy metabolism are anticipated from the finding that acute dietary intake modulates post-prandial succinate plasma levels by a mechanism that is dependent on intestinal glucose sensing and metabolic status [22]. Succinate is also a microbiota-derived metabolite with a key role in governing intestinal homeostasis [23]. Succinate levels are clearly elevated in inflammatory-related health conditions, including obesity and type 2 diabetes (T2D) [22, 24–26], and are also related to a microbiota dysbiosis signature [26]. Indeed, succinate has been validated as a surrogate biomarker of poor metabolic control in patients with obesity and T2D [22, 24, 26] and can predict diabetes remission in patients undergoing bariatric surgery [24]. To date, however, no study has investigated whether circulating succinate levels are associated with CVD risk, or whether it can be a biomarker of CVD risk in young adults [3–6].

Oxylipins are a large family of lipid-based metabolites derived from polyunsaturated fatty acids that differentially regulate inflammatory processes, representing a novel group of putative CVD risk biomarkers [27, 28]. Omega-3 oxylipins mainly exert anti-inflammatory and pro-resolving effects, whereas omega-6 oxylipins are mainly involved in pro-inflammatory processes [29, 30]. Interestingly, previous work has established a link between higher levels of circulating omega-6 oxylipins and an elevated pro-inflammatory status and CVD risk [31, 32], but little is known about the role of omega-3 oxylipins for CVD risk.

In the present study, we aimed to determine the relationship between plasma succinate levels and CVD risk in young adults. We examined whether succinate levels correlate with traditional and novel CVD risk factors (i.e., oxylipins) in a well-phenotyped cohort of young adults.

## Methods

### Participants

The present study was conducted within the framework of the ACTIvating Brown Adipose Tissue through Exercise (ACTIBATE) study [33], a randomized controlled trial designed to determine the effect of exercise on BAT activity (Clinical trials identifier: NCT02365129). Inclusion criteria were the following: to be sedentary (< 20 min moderate-to-vigorous physical activity on < 3 days/week), non-smoker, not taking any medication, and stable body weight over the last 3 months. Exclusion criteria were: diagnosis of diabetes, hypertension or any medical condition(s) that can interfere with or be aggravated by exercise, being pregnant, using medication (including antibiotics) that could affect energy metabolism or gut microbiota, and being frequently exposed to cold temperatures (e.g., indoors/outdoors workspace with low-temperatures, such as cold-storage works, ski/snow monitors, fieldwork during the winter sessions or low-temperature areas). All participants gave their informed consent and the study was approved by the Ethics Committee on Human Research of the University of Granada (no. 924), and Servicio Andaluz de Salud (Centro de Granada, CEI-Granada).

We selected participants from the ACTIBATE study with valid data for serum CVD risk factors, body composition, dietary intake, basal metabolic rate (BMR), cardiorespiratory fitness, brown adipose tissue (BAT) volume and activity, and with plasma samples available for succinate measurements. This resulted in a cohort of 100 young adults (65 women, 35 men; age 18–25 years) that were used in subsequent analyses. Of this cohort, 58 individuals had available fecal samples that were used in the fecal microbiota and composition analysis. Table 1 shows the descriptive characteristics of the participants as well as the plasma succinate levels measured at baseline.

**Table 1:** Characteristics of the individuals by tertiles of plasma succinate levels

	Plasma succinate tertiles			P
	Low (11.6–55.1 µM) n = 34	Intermediate (55.2–71.4 µM) n = 33	High (71.5–129.8 µM) n = 33	
Age (years)	21.7 ± 2.3	22.6 ± 2.1	21.6 ± 1.9	0.137
Sex (n, %)				0.777
Men	13 (38.2)	10 (30.3)	12 (36.4)	
Women	21 (61.8)	23 (69.7)	21 (64.6)	
Weight status (n, %)				0.097
Normal-weight	23 (67.7)	19 (57.6)	15 (45.5)	
Overweight	8 (23.5)	11 (33.3)	10 (30.3)	
Obese	3 (8.8)	3 (9.1)	8 (24.2)	
BMI (kg/m^2^)	24.2 ± 3.8	24.0 ± 3.8	26.1 ± 5.2	0.091
LMI (kg/m^2^)	14.8 ± 2.1	14.1 ± 2.2	15.1 ± 2.7	0.275
FMI (kg/m^2^)	8.1 ± 2.7	8.5 ± 2.6	9.6 ± 3.3	0.077
Body fat (%)	33.6 ± 7.4	35.8 ± 6.7	37.0 ± 8.1	0.170
VAT (g)	289* ± 146	346 ± 178	411* ± 201	**0.020**
Waist circumference (cm)	78.8 ± 11.9	80.9 ± 13.3	84.3 ± 14.7	0.247
Glucose (mg/dL)	86.5 ± 6.2	88.5 ± 6.5	87.3 ± 6.9	0.466
Insulin (μUI/mL)	7.4 ± 3.9	8.2 ± 3.9	9.6 ± 6.0	0.126
HOMA index	1.6 ± 1.0	1.8 ± 1.0	2.1 ± 1.6	0.152
Total cholesterol (mg/dL)	156.2 ± 25.0	162.6 ± 31	170.2 ± 34	0.230
HDL-C (mg/dL)	51.3 ± 9.7	52.4 ± 9.5	54.2 ± 15	0.808
LDL-C (mg/dL)	91.2 ± 23	94.3 ± 25	95.7 ± 29	0.907
Triglycerides (mg/dL)	68.2* ± 28	80.0 ± 40	111.8* ± 70	**0.002**
C-reactive protein (mg/L)	1.7* ± 2.2	2.1 ± 2.1	3.8* ± 5.1	**0.039**
SBP (mmHg)	114.1 ± 11	116.7 ± 11	120.4 ± 12	0.082
DBP (mmHg)	68.8* ± 7.7	71.4 ± 5.5	73.7* ± 7.6	**0.023**
Metabolic syndrome ATPIII (n, %)	0 (0)	1 (3)	4 (12.1)	0.068
Cardiorespiratory fitness (mL/kg/min)	44.9* ± 7.3	38.8* ± 7.7	40.6 ± 6.9	**0.004**
BAT volume (mL)	71.3 ± 47.3	71.0 ± 68.8	64.3 ± 56.2	0.825
BAT SUVmean	4.0 ± 2.1	3.6 ± 2.0	3.8 ± 1.8	0.794
Basal metabolic rate (kcal/d)	1335 ± 570	1518 ± 896	1407 ± 198	0.534
Energy intake (kcal/d)	1904 ± 463	1769 ± 397	1950 ± 497	0.115
Energy density intake (kcal/g/d)	1.5 ± 0.4	1.5 ± 0.3	1.4 ± 0.3	0.333
Fat intake (g/d)	84.8 ± 25.9	78.3 ± 26.4	85.7 ± 23.9	0.156
Protein intake (g/d)	77.8* ± 20.2	70.2* ± 14.6	76.4 ± 21.6	**0.003**
Carbohydrates intake (g/d)	202.4 ± 67.0	191.1 ± 64.4	214.2 ± 73.6	0.378

Significant P values (P < 0.05) are shown in bold

Data are presented as mean and standard deviation (SD), otherwise stated

*ATPIII* National Cholesterol Education Program Adult Treatment Panel III, *BAT* brown adipose tissue, *BMI* body mass index, *DBP* diastolic blood pressure, *FMI* fat mass index, *HDL-C* high density lipoprotein-cholesterol, *HOMA index* homeostatic model assessment, *LDL-C* low density lipoprotein-cholesterol, *LMI* lean mass index, *SBP* systolic blood pressure, *SUV* standardized uptake value, *VAT* visceral adipose tissue

P from one-way analysis of variance, or from chi-square test (categorical variables). Plasma succinate levels are computed as tertiles

*Symbols indicates significant differences between groups (P < 0.05) after Bonferroni correction for multiple comparisons

### Procedures

All data were collected at the same hour of the day, but on different days within a period of 3 weeks. Participants commuted to the research center by car, bus, or motorcycle, and all reported to have slept as usual and refrained from stimulant beverages and any moderate physical activity in the previous 24 h, or any vigorous physical activity in the 48 h prior to each visit. Participants remained still (either lying down or sitting) during the assessments. Self-reported menstrual cycle phase of female participants was recorded at each visit. All data, material and methods are available on request. Detailed procedures can be found as Additional file 1. 

### Classification of individuals into metabolic healthy overweight-obese and metabolic unhealthy overweight-obese groups

Individuals were categorized as metabolic healthy overweight/obese (MHOO; n = 27) or metabolic unhealthy overweight/obese (MUOO; n = 16) as described [34]. The MHOO group included individuals with a body mass index (BMI) ≥ 25 kg/m^2^ and without any of the following cardiovascular risk factors: (i) serum HDL-C < 40 mg/dL for men and 50 mg/dL for women; (ii) serum triglycerides > 150 mg/dL; (iii) systolic blood pressure > 130 mmHg or diastolic blood pressure > 85 mmHg; or (iv) serum glucose > 100 mg/dL. The MUOO group included individuals with BMI ≥ 25 kg/m^2^ and presenting with at least one of the aforementioned cardiovascular risk factors.

### Statistical analysis

Data are presented as means ± standard deviations (unless otherwise stated). Plasma succinate levels were computed as tertiles (low, intermediate or high levels) using the function “Visual Binning” with SPSS (SPSS v. 22.0, IBM SPSS Statistics, IBM Corp. Armonk, NY). For descriptive characteristics, categorical and continuous variables were used according to plasma succinate levels. Differences in categorical variables between groups were analyzed by chi-square tests, whereas differences in continuous variables between groups were analyzed by one-way analyses of variance. The level of significance between groups was set at P < 0.05, after Bonferroni correction for multiple comparisons. Serum levels of cardiovascular risk parameters and plasma omega-3 and omega-6 oxylipins levels were log10 transformed to achieve a normal distribution. Plasma succinate levels followed a normal distribution and were not transformed. The plasma succinate fold-change value was used to perform interaction network pathway analyses of plasma omega-3 and omega-6 oxylipins, computed as the ratio of the mean values of the two compared groups (i.e., high vs. low fold change = average succinate levels (high tertile)/average succinate levels (low tertile). Fold-change differences were analyzed with an unpaired t-test. The sex distribution in the classification of MHOO and MUOO individuals was not similar; therefore, to study whether plasma succinate levels were different between groups the analyses were adjusted by sex as a covariate. No sex interaction was detected in the other analyses (all P > 0.05). Figure 3 and Additional file 6: Fig. S1 were built using GraphPad Prism version 8.0.0 for Windows (GraphPad Software, San Diego, CA). Figure 2 was built using Cytoscape software version 3.7.0 for Windows (Boston, MA) [35].

## Results

The characteristics of the participants included in the study are shown in Table 1. We found a great variability in plasma succinate levels in the cohort (11.6–129.8 µM; amplitude range, 118.2 µM) (Additional file 6: Fig. S1). Given this broad range, we used the low (11.6–55.1 µM), intermediate (55.2–71.4 µM), and high (71.5–129.8 µM) plasma succinate tertiles for subsequent analyses (Table 1).

### Young adults with higher plasma succinate levels have higher visceral adiposity and an adverse cardiovascular profile

Participants in the highest tertile of succinate had significantly higher visceral adipose tissue (VAT) mass (+ 42.5%), serum triglyceride levels (+ 63.9%), serum C-reactive protein levels (+ 124.2%), and diastolic blood pressure (+ 5.5%) than peers in the lowest tertile (Table 1). By contrast, cardiorespiratory fitness levels were significantly higher in the lowest tertile (up to + 15.7%) than in the intermediate tertile (Table 1). No significant differences were found in dietary energy and macronutrients intake, dietary energy density parameters, BMR levels or BAT parameters across the three tertiles (Table 1).

### Plasma succinate levels are not associated with fecal microbiota composition and diversity

**Fig. 1:**
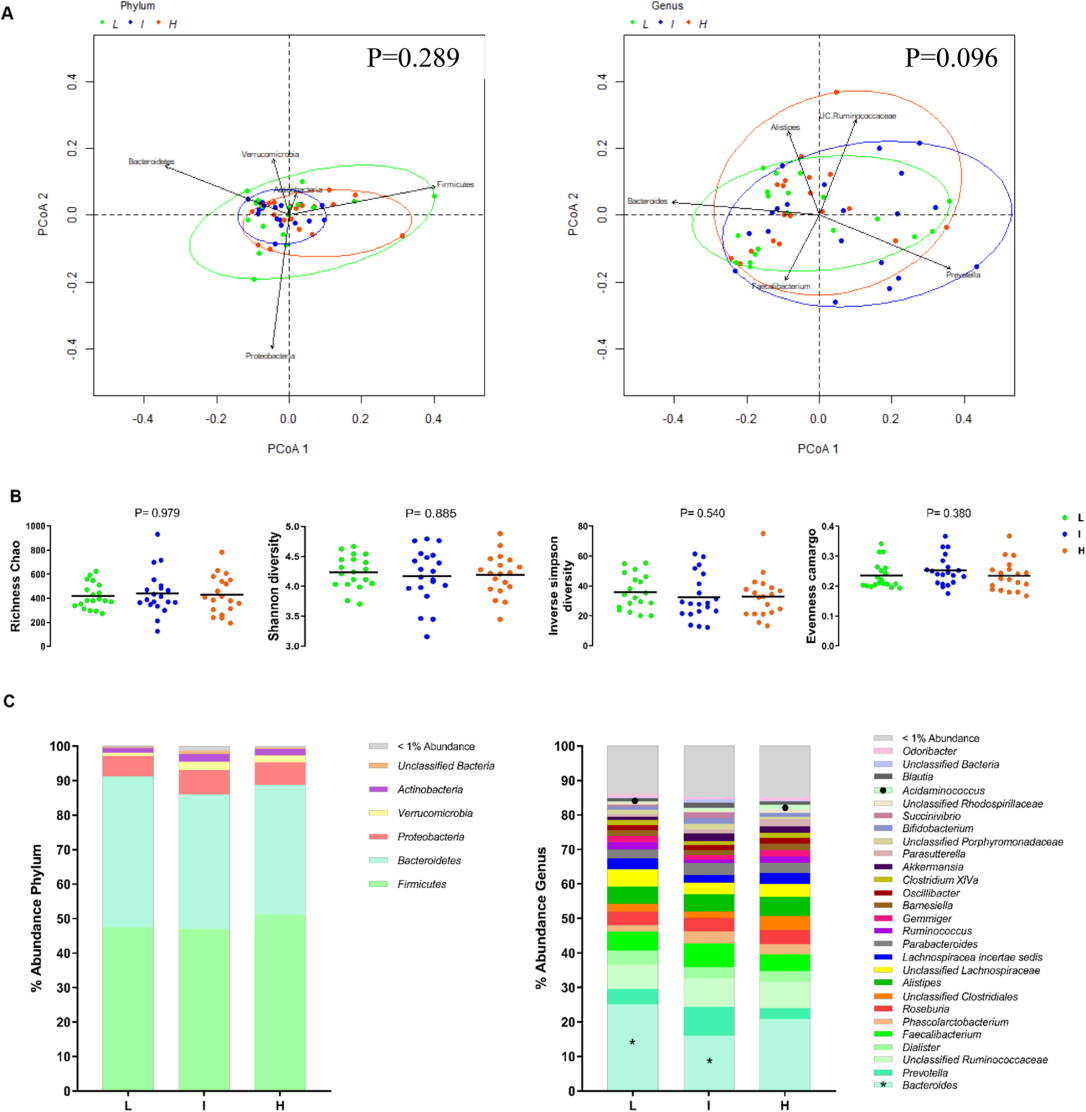
Fecal microbiota diversity and composition by tertiles of plasma succinate (n = 58). L: Low succinate plasma concentration (11.6–57.3 µM); I: Intermediate succinate plasma concentration (57.3–75.3 µM); H: High succinate plasma concentration (75.4–129.8 µM). **A** Principal Coordinate Analysis (PCoA) plot of the first two principal coordinates at phylum and genus level, categorized by circulating succinate levels. Genus PCoA only shows PCoA analyses done using Bray–Curtis dissimilarity. Ellipses represent the 95% confidence intervals (package, vegan, R version 3.6). **B** Differences between the circulating succinate tertiles in fecal microbiota diversity indexes (richness Chao, Shannon, inverse of Simpson, and evenness Camargo). Kruskal–Wallis test (P < 0.05) was used to test for each pairwise comparison. **C** Relative abundance of the fecal microbiota at phylum (left panel) and genus level (right panel) according to circulating succinate levels. Stacked bar represented percentage abundance. The symbol (asterisk) means statistical significance differences between Low and Intermediate levels, whereas the symbol (filled circle) means statistical significance differences between low and high levels, determined by Kruskal–Wallis test, corrected for multiple comparisons FDR (P < 0.05)

Analysis of the fecal microbiota in young adults revealed no association between succinate levels and beta or alpha diversity (all P ≥ 0.380 Fig. 1a, b). Similarly, no associations were found between succinate levels and relative abundances at the phylum level (all P > 0.05; Fig. 1c, left panel). Nonetheless, we found that individuals in the lowest tertile of plasma succinate had a higher relative abundance of *Bacteroides* (+ 56.9%) and a lower relative abundance of *Acidaminococcus* (− 93.8%) than individuals in the intermediate and highest tertiles, respectively (all P ≤ 0.01; Fig. 1c*,* right panel). No significant differences were observed in the relative abundance of the species belonging to *Bacteroides* and *Acidaminococcus* genera across the succinate tertiles (all P > 0.05; Additional file 3: Table S2). Likewise, no associations were found between succinate levels and succinate-producing or succinate-consuming species (all P > 0.05; Additional file 4: Table S3 and Additional file 7: Fig. S2a, b).

### Plasma succinate levels are associated with pro-inflammatory omega-6 oxylipins

**Fig. 2:**
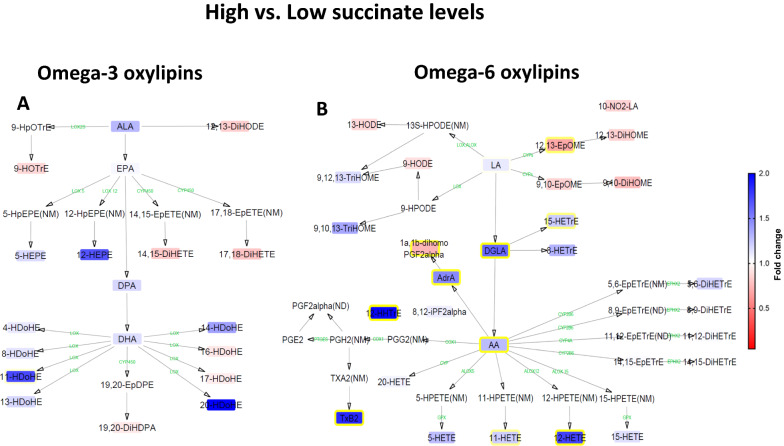
Interaction network pathway analysis of circulating omega-3 and omega-6 oxylipins (n = 98). The networks depict the differences of each lipid mediator between high versus low tertiles of plasma succinate levels. Compounds that were not detected (ND) or not measured (NM) with the LC–MS/MS method are shown without boxes. Blue boxes with yellow borderlines indicate that this lipid mediator was higher in high versus the low succinate group, whereas red boxes with yellow borderlines indicate that this lipid mediator was lower in the comparison. Boxes without yellow borderlines indicate that this lipid mediator did not significantly change in the comparison. Fold-change values are depicted according to the legend bar on the right side. Intermediate enzymes are represented in green, and they were not measured. Comparisons are performed with independent t-test analysis (log10 transformed values) and P < 0.05

Given the clear link between plasma succinate and some pro-inflammatory markers, such as serum C-reactive protein and serum triglycerides (Table 1) [36], and the lack of associations between plasma succinate levels and classical inflammatory markers (i.e., IL-6, TNF-α or IFN-γ; data not shown), we extended our investigation to the fatty acid-derived oxylipins. No significant differences were observed in plasma omega-3 oxylipins when comparing the high versus low succinate tertiles (Fig. 2a) (abbreviations are detailed in Additional file 2: Table S1). However, individuals in the highest succinate tertile had significantly higher plasma concentrations of omega-6 oxylipins than individuals in the lowest tertile, including the omega-6 fatty acids DGLA (+ 61.1%), AdrA (+ 47.4%) and AA (+ 28.7%), as well as several of their downstream products, as revealed by interaction network pathway analysis (Fig. 2b). In addition, individuals in the highest succinate tertile had significantly lower levels of the omega-6 oxylipins 12,13-EpOME (− 30.7%) and 1a,1b-dihomo PGF_2alpha_ (− 25.4%). With respect to DGLA metabolism, the hydroxy-trienoic acid product resulting from 15-lipoxygenation of DGLA (15-HETrE), was found to be significantly higher in concentration in individuals in the highest succinate tertile than in those in the lowest succinate tertile (+ 21.8%). The concentration of other downstream AA-derived oxylipins was also significantly higher in individuals in the highest tertile of plasma succinate, including 11-HETE (+ 20.4%), 12-HETE (+ 74.9%), 12-HHTrE (+ 97.9%) and TxB2 (+ 84.8%). Only small differences in oxylipin levels were observed when comparing high versus medium succinate tertiles (Additional file 8: Fig. S3A), but the differences observed between medium versus low succinate tertiles (Additional file 8: Fig. S3B) resembled the differences between high and low succinate tertiles.

### Young adults with metabolically unhealthy overweight/obesity have higher plasma succinate levels than their metabolically healthy counterparts

**Fig. 3:**
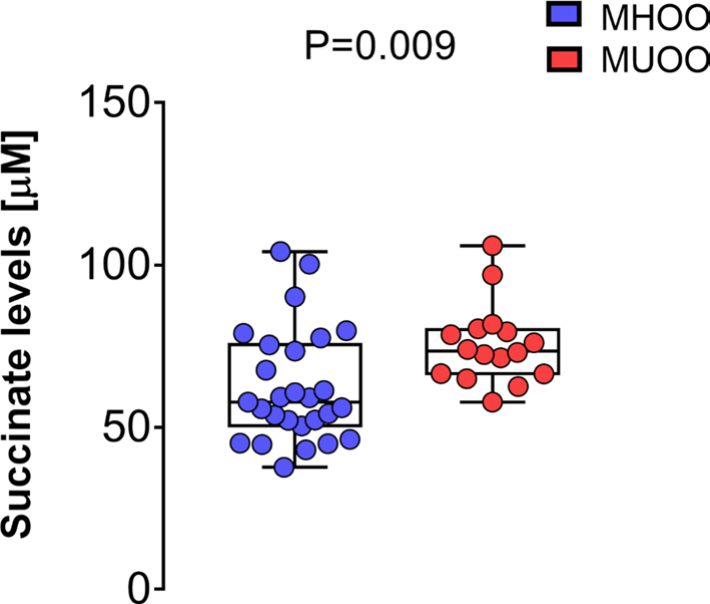
Comparisons between plasma succinate levels in metabolic healthy overweight/obese (MHOO, n = 27) and metabolic unhealthy overweight/obese (MUOO, n = 16) young adults. P value obtained from one-way analysis of variance adjusted for sex

To gain more insight into the potential role of plasma succinate as an early marker of cardiovascular risk, we subcategorized the overweight/obese individuals of the cohort (43% of our population) as healthy (MHOO; n = 27) or unhealthy (MUOO; n = 16) based on their cardiovascular profile. Individuals in the MUOO group had higher BMI (+ 7.2%), FMI (+ 3.1%), VAT mass (+ 24.3%), fasting glucose (+ 7.6%), insulin (+ 57.9%), homeostatic model assessment index (+ 71.5%), total cholesterol (+ 15.4%), triglycerides (+ 119.8%), and systolic (+ 8.2%) and diastolic (+ 10.1%) blood pressure than peers in the MHOO group, and had lower HDL-C levels (− 16.1%) (Additional file 5: Table S4). Moreover, plasma succinate levels were significantly higher (+ 21.3%) in the MUOO group (75.5 ± 12.3 µM) than in the MHOO group (62.3 ± 17.4 µM) (P = 0.009; Fig. 3).

## Discussion

Here, we demonstrate for the first time to our knowledge that plasma succinate is associated with VAT mass, serum triglycerides and C-reactive protein levels, and diastolic blood pressure in young adults. Likewise, individuals with higher levels of plasma succinate have higher levels of plasma omega-6 oxylipins, which are linked to increased pro-inflammatory status and, accordingly, elevated CVD risk [31, 32]. By contrast, plasma succinate levels are not associated with BAT or with fecal microbiota composition and diversity. Interestingly, individuals who are metabolically unhealthy with overweight/obesity have higher plasma succinate levels than their metabolically healthy counterparts. Collectively, our findings suggest that plasma succinate is candidate biomarker of cardiovascular risk in young adults. Further studies are, nevertheless, needed to unravel the underlying mechanisms that may explain these associations.

Investigations of the association of plasma succinate levels with body composition are scarce. We recently showed that plasma succinate levels are positively associated with BMI in middle-aged and elderly adults with obesity and T2D [24, 26]. In the present study, we found that young adults with higher succinate levels have higher VAT mass, an established marker of elevated CVD risk [37–39]. Visceral fat depots are linked to metabolic dysfunction through increased mitochondrial oxidative stress, a main driver of cellular insulin resistance [40]. Interestingly, succinate dehydrogenase (SDH) activity is a potential source of reactive oxygen species (ROS) under specific conditions, including obesity [41–43], suggesting an adverse influence of SDH in obesity. In this context, some of the metabolic improvements observed after bariatric surgery are, in part, due to the restoration of SDH activity in VAT, which concurs with the weight loss caused by the surgery [44]. It is thus reasonable to propose that a fraction of the plasma succinate could originate in (and be secreted from) VAT depots, through SDH activity, contributing to the positive association between plasma succinate levels and VAT mass.

The gut microbiota plays a key role in regulating host metabolism, and specific signatures of gut microbiota composition have been associated with obesity, insulin resistance, and T2D [45]. Succinate is a primary cross-feeding metabolite between gut-resident microbes, which is important to preserve a healthy gut microbiota [23]. We recently demonstrated in middle-aged adults that the fecal microbiota is a putative contributor of circulating succinate levels in some health conditions such as obesity [26]. Our previous data also support the notion that succinate is a marker of microbiota dysbiosis, as intestinal permeability positively correlates with circulating succinate in individuals with obesity [26]. Nonetheless, we found no association between plasma succinate and succinate-producing or succinate-consuming bacteria species in the present analysis. Differences in the age and metabolic status of the cohorts, or even the moderate sample size of the present study (n = 100), may partly explain these findings.

We previously showed that circulating succinate is regulated nutritionally [22], and it is known that SUCNR1 has an intracellular anti-lipolytic function [46, 47]. Studies in mice have recently demonstrated that succinate uptake stimulates uncoupling protein 1 (UCP1)-dependent thermogenesis in brown adipocytes via ROS production, protecting against diet-induced [19]. Our present study, however, failed to show a significant association between systemic succinate and BAT volume or activity. Given the cross-sectional nature of our study, we cannot draw conclusions on a potential role of succinate in human BAT thermogenesis. Additionally, neither plasma succinate levels nor BMR were associated with parameters of energy intake/consumption in our young cohort. Nonetheless, plasma succinate levels were inversely associated with cardiorespiratory fitness. In this line, succinate has recently emerged as an important player in muscle adaptation in response to exercise [20, 48]. While the observational design of our present study does not enable us to infer causality, it adds to the increasing body of evidence on the role of succinate as a possible mediator of the cardiovascular benefits of exercise. Cardiorespiratory fitness is also recognized as a relevant risk factor associated with adverse cardiovascular health and poor prognosis [49], and a lower prevalence of metabolic syndrome has been reported in people with better cardiorespiratory fitness [50]. This may fit with our finding that the MUOO group had significantly higher succinate levels than the MHOO group, which also supports previous data showing higher succinate levels in obese individuals with T2D than in their healthy obese counterparts [26]. Altogether, these results strengthen the hypothesis that high plasma succinate levels are linked to an impaired metabolic status.

Young adults with high plasma succinate levels also had higher plasma levels of C-reactive protein and pro-inflammatory oxylipins. Omega-6 oxylipins are key metabolites in pro-inflammatory processes and are closely linked to the progression of obesity and to cardiovascular risk [31, 32], whereas omega-3 oxylipins usually have opposing effects [31, 32]. Individuals with high succinate levels had higher circulating AA, a pivotal fatty acid precursor in a plethora of inflammatory processes [51], in addition to several AA downstream metabolites involved in pro-inflammatory processes [52, 53]. Specifically, we observed that circulating succinate levels were related to the omega-6 oxylipins 11-HETE, 12-HETE, 12-HHTrE, and 15-HETrE. Previous research has linked 5-HETE and 11-HETE to obesity [54–56], whereas the auto-oxidative product 15-HETrE has been associated with a high risk of cardiovascular events [57]. Of particular note is the high level of TxB2 in individuals with high succinate, which is reported to increase in patients with coronary atherosclerosis [58]. In addition to an increase in AA products in individuals with high succinate levels, we also observed an increase in the precursor DGLA, which has been previously associated with obesity and insulin resistance [59]. Likewise, the AA metabolic product AdrA, which was higher among participants in the highest succinate tertile, has been shown to be directly associated with the risk of all-cause mortality [60]. As many of the enzymes (i.e., cyclooxygenases and lipoxygenases) involved in oxylipin metabolism are shared by both omega-3 and omega-6 oxylipins [61], it is not surprising to find an asymmetric pattern in the omega-3 and omega-6 oxylipins profile in the high versus low succinate groups.

### Strengths and limitations

A major strength of the present study is the well-characterized population, including detailed measurements of body composition, BAT, and novel markers of the cardiometabolic profile such as oxylipins, which allow us to gain new insight into the inflammatory status of the individuals compared with classical inflammatory markers (i.e., IL-6, TNF-α or IFN-γ). A major limitation of the study is its cross-sectional design, and no causality can be established. Another limitation is the limited sample size for multivariate statistical analyses. Finally, although ^18^F-FDG uptake is the current gold-standard for BAT quantification, it also has limitations in the assessment of BAT metabolic activity and volume [62].

## Conclusion

Our study reveals that plasma succinate levels are linked to a specific pro-inflammatory omega-6 signature pattern and higher VAT levels, and might be useful as a novel clinical tool to identify young individuals at higher CVD risk, allowing the implementation of effective preventive treatment. Plasma levels of succinate seem to reflect the cardiovascular status of young adults, supporting its potential as a biomarker of CVD risk. Prospective studies are needed to confirm its clinical relevance and predictive value as a CVD risk biomarker.

## Supplementary Information

**Additional file 1.** Supplementary methods.

**Additional file 2: Table S1.** List of metabolites analyzed by LC-MS/MS.

**Additional file 3: Table S2.** Relative abundance (%) of species belonging to the Bacteroides and Acidaminococcus genera by tertiles of plasma succinate (n = 58).

**Additional file 4: Table S3.** Relative abundance (%) of succinate-producing and -consuming species previously described by Serena C. et al. by tertiles of plasma succinate (n = 58).

**Additional file 5: Table S4.** Characteristics of metabolically healthy overweight/obese (MHOO, n = 27) and metabolically unhealthy overweight/obese (MUOO, n = 16) individuals.

**Additional file 6: Fig S1.** Waterfall plot showing plasma succinate levels per individual (n=100). Each bar represents a single individual.

**Additional file 7: Fig. S2.** Differences at family (A) and genus (B) levels by tertiles of plasma succinate levels (n = 58).

**Additional file 8: Fig. S3.** Interaction network pathway analysis of circulating omega-3 and omega-6 oxylipins (n = 98)

## Data Availability

The data that support the findings of this study are available from the corresponding author upon reasonable request.

## Abbreviations

BAT: Brown adipose tissue
BMI: Body mass index
BMR: Basal metabolic rate
CVD: Cardiovascular disease
^18^F-FDG: ^18^F-fluorodeoxyglucose
FMI: Fat mass index
LC–MS/MS: Liquid chromatography–tandem mass spectrometry
MHOO: Metabolically healthy overweight and obese individuals
MUOO: Metabolically unhealthy overweight and obese individuals
PET–CT: Positron emission tomography–computed tomography
ROS: Reactive oxygen species
SUCNR1: Succinate receptor 1
SDH: Succinate dehydrogenase
SUV: Standardized uptake value
T2D: Type 2 diabetes
TCA: Tricarboxylic acid
UCP1: Uncoupling protein 1
VAT: Visceral adipose tissue
