# Supplementary material for: Elevated plasma succinate levels are linked to higher cardiovascular disease risk factors in young adults
Source: Cardiovasc Diabetol. 2021 Jul 27;20:151. doi: 10.1186/s12933-021-01333-3 (PMC8314524; doi:10.1186/s12933-021-01333-3)
Supplement: Supplementary file 1 — Additional file 1. Supplementary methods. [file 12933_2021_1333_MOESM1_ESM.docx]

**ADDITIONAL FILE 1**

**SUPPLEMENATARY METHODS**

**Anthropometry, basal metabolic rate, and dual-energy X-ray absorptiometry**

On the first visit, participants arrived at 08:15 AM (after a 12-h overnight fast, with a standardized dinner the evening before). Waist circumference was measured twice at the minimum perimeter area with a measuring tape (mm precision) and the mean value was calculated. For those with abdominal obesity, waist circumference was measured just above the umbilicus (horizontal plane). Body mass and height were measured (no shoes, light clothing) using a model 799 Seca scale and stadiometer (Seca, Hamburg, Germany). After having urinated, participants put on standardized clothes (clothing insulation value: 0.20) and entered a warm room (22.8 ± 0.9°C; 43.8 ± 6.7% humidity). Basal metabolic rate (BMR) was measured during 30 min while lying down on a bed using a CCM Express or Ultima CardiO2 metabolic cart (Medical Graphics Cardiorespiratory Diagnostics St Paul, MN) [1,2], according to methodological recommendations [3]. We selected the average of the most stable 5-min period, as it was the most accurate estimation of the individuals’ BMR [1]. Body fat mass, lean body mass and visceral adipose tissue (VAT) were measured by whole-body dual-energy X-ray absorptiometry (HOLOGIC, Discovery Wi, Marlborough, MA). Body mass, lean mass, and fat mass indices were calculated as kg/m^2^.

**Positron emission tomography-computed tomography scanning and analysis**

On the second visit, participants arrived in a fasted condition (≥6 h) and were placed in a cool room (19.5–20°C) wearing a water-perfused cooling vest (Polar Products Inc., Stow, OH) and the same standardized clothes as on visit 1. Water temperature was progressively reduced until shivering occurred (self-reported and visually observable). The water temperature at the onset of shivering was recorded as the shivering threshold (5.4 ± 2.2°C for men and 6.3 ± 2.2°C for women; common range for both sexes is 3.9–12.2°C).

At 48–72 h after the shivering threshold test, on visit 3, the participants were placed in a cool room (19.5–20°C) with the cooling vest temperature set at 4°C above their individual shivering threshold. After 1 h of cold exposure with a cooling vest 4°C above their individual shivering threshold and the room temperature at 19.5–20°C, they received an intravenous injection of ∼185 MBq ^18^F-FDG while the water temperature was increased by 1°C. The PET-CT scan was performed one hour after the injection, and scans were analyzed using the Beth Israel plug-in for FIJI software [4], in agreement with the methodological recommendations [5] and following a protocol described elsewhere [6,7]. PET-CT images from cervical vertebra 1 to thoracic vertebra 6 (approximately) were obtained. To assess BAT volume and ^18^F-FDG uptake, we selected voxels with a radiodensity between −190 and -10 Hounsfield Units and an ^18^F-FDG uptake above the individualized standardized uptake value (SUV) threshold of 1.2/(lean body mass/body mass) [5]. Based on this information, BAT volume and ^18^F-FDG uptake (calculated as SUV mean) parameters were obtained following the BARCIST 1.0 recommendations [5].

**Cardiorespiratory fitness**

On the fourth visit, individuals arrived in fasting conditions (3–5 h) having refrained from drinking coffee/tea during the testing day or the day before. Neither vigorous exercise (48 h before) nor moderate exercise (24 h before) was allowed prior to the assessments. A treadmill maximum-exercise test employing an H/P/Cosmos Pulsar treadmill (H/P/Cosmos Sports & Medical GmbH, Nussdorf-Traunstein, Germany) was performed according to a modified Balke protocol [8]: 1-min warm-up at 3 km/h, followed by 2 min at 4 km/h, and 1 min at 5.3 km/h. Subsequently, the treadmill slope was increased by 1% each minute until volitional exhaustion was reached. Respiratory gas exchange was monitored with a CPX Ultima CardioO_2_ system (Medical Graphics Corp., St Paul, MN) with a facemask, model 7400 (Hans Rudolph Inc., Kansas City, MO), and a preVent™ metabolic flow sensor (Medical Graphics Corp.) [1]. Carbon dioxide production (VCO_2_) was assessed using a non-dispersive infra-red sensor, and oxygen consumption (VO_2_) was measured using a galvanic fuel cell [1]. Maximum VO_2_ (VO_2_max) was defined as a respiratory exchange ratio of ≥1.1, once a VO_2_ plateau was reached, with a heart rate within 10 beats/min of the individuals’ age-predicted maximum (209-0.73×age) [9]. VO_2_ max was calculated relative to body mass [10].

**Cardiovascular disease risk factors and plasma succinate analysis**

During the fifth visit, blood samples were drawn from the antecubital vein in the morning (8.00–9.00 A.M) after overnight fasting (>10 h), under resting conditions. Blood samples were collected in Vacutainer Tubes^®^, which were immediately centrifuged, and serum (obtained with Vacutainer^®^ SST™ II Advance tubes) and plasma (obtained with Vacutainer^®^ Hemogard™ tubes, containing potassium salt of ethylenediamine tetra-acetic as anticoagulant) aliquots were stored at -80°C until analyses. Serum samples were used for cardiovascular risk factor analyses, whereas plasma samples were used to determine succinate and omega-3 and omega-6 oxylipin concentrations.

Glucose was measured in an AU5832 biochemical analyzer (Beckman Coulter, Brea, CA) using a Beckman Coulter reagent (#OSR6521) and insulin was measured in a DXI analyzer (Beckman Coulter) using a Beckman Coulter chemiluminescent reagent (#33410). These values were used to calculate the homeostatic model assessment (HOMA) index of insulin resistance [11]. Total cholesterol, triglyceride, and high-density lipoprotein-cholesterol (HDL-C) serum levels were measured in the AU5832 analyzer using the Beckman Coulter reagents #OSR6116, OSR60118 and OSR6187, respectively. Low-density lipoprotein-cholesterol (LDL-C) levels were subsequently calculated using the Friedewald formula: (total cholesterol) − (HDL-C) − 0.45 * (triglycerides). [12] C-reactive protein was also measured in an AU5832 analyzer with the reagent #OSR6299.

Plasma succinate levels were measured using the EnzyChrom^TM^ Succinate Assay Kit (BioAssay Systems, Hayward, CA). The assay sensitivity was 12 µM and the intra- and inter-assay coefficients of variance were <3.50% and 6.95%, respectively, and the accuracy ranged from 1 to 11.5% error [13,14].

Systolic and diastolic blood pressure was measured with an automatic sphygmomanometer (Omrom M2; Omron Healthcare, Kyoto, Japan). Measurements were repeated on three different days and the averages were calculated.

The prevalence of metabolic syndrome was calculated according to the National Cholesterol Education Program Adult Treatment Panel III (ATP III) criteria [15] .Participants were considered to have metabolic syndrome if they had three or more of the following risk factors: waist circumference ≥102 cm for men and ≥88 cm for women; triglycerides ≥150 mg/dL; HDL-C <40 mg/dL for men and <50 mg/dL for women; systolic blood pressure ≥130 mmHg or diastolic blood pressure ≥85 mmHg; glucose >110 mg/dL.

**Fecal microbiota analysis**

On the sixth visit, a fecal sample (50–60 g) was collected from a sub-cohort of n=58 participants using a sterilized plastic container. Samples were transported in a portable cooler at 4°C to the laboratory and stored at -80°C until DNA extraction. Fecal samples were homogenized in a Stomacher® 400 (A. J. Seward and Co. Ltd., London, UK) and DNA extraction and purification were performed with a commercial kit (QIAamp DNA Stool Mini Kit, QIAGEN, Barcelona, Spain).

DNA was quantified using a NanoDrop ND1000 spectrophotometer (Thermo Fisher Scientific, DE) and quality was evaluated according to the A260/280nm and A260/230nm absorbance ratios.

Purified DNA was amplified by PCR targeting the V3 and V4 hypervariable regions of the bacterial 16S rRNA gene by using the following primer pairs, 16S Amplicon Forward Primer: 50TCGTCGGCAGCGTCAGATGTGTATAAGAGACAGCCTACGGGNGGCWGCG, and 16S Amplicon Reverse Primer: 50GTCTCGTGGGCTCGGAGATGTGTATAAGAGACAGGACTACHVGGGTATCTAATCC. [16] PCR assays were conducted in a final volume of 25 µL, consisting of 12.5 µL 2× KAPA HiFi Hotstart ready mix (KAPA Biosystems, Woburn, MA), 5 µL forward primer (1 µM), 5 µL reverse primer (1 µM), 2.5 µL DNA (10 ng), with the following PCR program: 1) denaturation (95ºC, 3 min); 2) 8 cycles of denaturation (95ºC, 30 s); annealing (55ºC, 30 s) and elongation (72ºC, 30 s); 3) final extension (72ºC, 5 min). Next, AMPure XP beads (Beckman Coulter, Indianapolis, IN) were used to purify the 16S V3 and V4 amplicons. A PCR indexing step was then performed, which attaches dual indices and Illumina sequencing adapters using the Nextera XT Index Kit (Illumina, San Diego, CA). The PCR conditions were as follows: 1) 95ºC, 3 min; 2) 8 cycles of 95ºC, 30 s; 3) 55ºC, 30 s; 4) 72ºC, 30 s; 5) 72ºC, 5 min; 6) hold at 4ºC. Pooled PCR products were puriﬁed using AMPure XP beads (Beckman Coulter) before quantification. Finally, the amplicons were sequenced at MiSeq (Illumina) using paired-end (2×300 nt) Illumina MiSeq sequencing system (Illumina).

Merging and pre-clustering of raw sequences was conducted using the “DADA2” [17] package in R [18], allowing differences in 2 nucleotides (so-called phylotypes), which were filtered according to a threshold for mean abundance of 0.001% and a sequence length 240 pb before the analysis. A total of 11,659,014 paired-end reads were obtained with an average of 126,728±33,395 reads per sample. All samples were above the 10,000 reads cut-off. Samples were standardized to an equal size of 30,982 reads using the “PHYLOSEQ” [19] package in R [18], obtaining a total of 11,158 phylotypes.

The “CLASSIFIER” function from the Ribosomal Database Project (RDP) was used for assigning taxonomic affiliation of phylotypes, according to the naive Bayesian classification [20] by using a pseudo-bootstrap threshold of 80%. A total 209 genera belonging to 16 different phyla were obtained. To further determinate the annotation of phylotypes (species assignments), the “SEQMATCH” function from RDP [21] was employed to define the discriminatory power of each sequence read; annotation was conducted according to previously published criteria [22]. Microbial communities were analyzed from phylum to species, calculating relative abundances expressed as percentages for use in subsequent analyses. Only the data for abundances higher than 1% relative abundance were represented at phylum and genus level according to the study variables.

Beta and alpha diversity metrics, and fecal microbiota composition, were then determined and used in the subsequent analyses. Beta diversity indicates differences in microbial community composition between individuals [23], whereas alpha diversity indicates the number of different phylotypes and relative abundances within a given individual [24]. Alpha diversity was calculated based on the Chao richness, inverse Simpson, Camargo’s evenness, and Shannon indices with the “MICROBIOME” [25] package in R software [18]. Chao richness estimates the diversity according to the number of different phylotypes identified in the community [26]; Shannon diversity increases as both the richness and the evenness of the community increase [27]; the inverse of Simpson diversity is calculated from classical Simpson diversity and indicates richness in a community with uniform evenness [28]; and Camargo’s evenness indicates the equitability of phylotypes frequencies in the community [29].

Data are presented as means ± standard deviations unless otherwise stated. Normality of all variables was assessed using the D’Agostino & Pearson omnibus with GraphPad Prism version 8.0.0 for Windows (GraphPad Software, San Diego, CA). Since variables were non-normally distributed, non-parametric tests were used for all analyses. The cohort was divided into tertiles according to circulating succinate levels (low, intermediate or high groups) using the Statistical Package for the Social Sciences v.22.0 (IBM SPSS Statistics, IBM Corporation, Chicago, IL). The “VEGAN” [30] R package was used for calculating the data matrix comprising the relative abundances at phylum and genus levels using the Bray-Curtis algorithm [31] for measuring beta diversity. Samples were ordinated by principal coordinate analysis. Significance level threshold was set at P<0.05. R software (V.3.6.0) [18] and GraphPad Prism were also used for plots. Beta diversity was measured quantitatively for relative abundance higher than 0.5% by permutational multivariate analysis of variance (PERMANOVA) based on Bray-Curtis [31] dissimilarity, with Past3 [32]. The Kruskal-Wallis test was used for the assessment of significant differences in gut microbiota composition and alpha diversity. P-values were corrected by the two-stage step-up method of Benjamini, Krieger and Yekutieli multiple comparison by controlling the False Discovery Rate (FDR).

**Dietary recalls**

Regular dietary energy intake was estimated using three non-consecutive 24-h dietary recalls, one of which was on a non-working day. Participants were interviewed by dietitians who recorded all food items and drinks that the individuals consumed on the day prior to the interview. The methodology has been extensively described elsewhere [33]. In brief, a book with pictures of different food servings and sizes was used to help participants estimate the amount of food consumed. EvalFINUT^®^ software (http://www.finut.org/evalfinut/) was used to obtain the nutritional composition of the diet, which was used to obtain dietary energy and macronutrients intake, and dietary energy density parameters. Consumption of water and salt was not recorded. Participants were not informed in advance when their diet was going to be registered.

**Determination of plasma omega-3 and omega-6 oxylipins**

Plasma levels of omega-3 and omega-6 oxylipins were measured and analyzed using a targeted metabolomics approach with liquid chromatography-tandem mass spectrometry (LC-MS/MS), as described elsewhere [34]. Using this LC-MS/MS method, 83 oxylipins were detected and relatively quantified (**Table S1**).

Oxylipins were extracted using liquid-liquid extraction [34]. Briefly, 150 µL of plasma was transferred into a 1.5 mL-Eppendorf tubes and was spiked with 5 µL of a solution of butylated hydroxytoluene (0.4 mg/mL) and 10 µL of a deuterated internal standard mix. Next, 150 µL of a buffer solution (0.2 M citric acid and 0.1 M disodium hydrogen phosphate) were added, followed by the addition of 1000 µL of the extraction solvent methyl tert-butyl ether and butanol (50:50, *v/v*). Samples were mixed for 5 min with a bullet blender (Next Advance, Averill Park, NY), and then centrifugated (16,000 *g*, 10 min, 4°C). After the centrifugation step, 900 µL of the upper layer was transferred to a new 1.5 mL Eppendorf tube. Samples were evaporated to dryness using a SpeedVac system prior to reconstitution in 50 µL of a solution of methanol:acetonitrile (70:30, *v/v*). The resulting solution was centrifuged (16,000 g, 10 min, 4°C), prior to the collection of 40 µL of the supernatant, which was transferred into glass vials for injection in the LC-MS/MS system.

The extracted samples were analyzed using a Shimadzu LC system (Shimadzu Corporation, Kyoto, Japan), coupled to a SCIEX QTRAP 6500+ mass spectrometer (SCIEX, Framingham, MA). Separation was performed using a BEH C18 column (50 mm × 2.1 mm, 1.7 μm) from Waters Technologies (Milford, MA) kept at 40°C. The mobile phase consisted of 0.1% acetic acid in water (A), 0.1% acetic acid in acetonitrile/methanol (90:10, *v/v*, B), and 0.1% acetic acid in isopropanol (C). Ionization was performed using electrospray ionization in negative mode. For the MS/MS acquisition, selected reaction mode (SRM) was employed. SRM transitions were individually optimized for targeted analytes and respective internal standards using standard solutions. The list of internal standards is shown in.

For each target compound detected, the ratio between its peak area and the peak area of its corresponding internal standard was calculated using SCIEX OS Software. Quality control (QC) samples (i.e., blank plasma samples) were used to evaluate the quality of the data and to correct for between-batch variations, using the in-house developed mzQuality workflow (available at http://www.mzQuality.nl). [35] Relative standard deviations (RSDs) of the peak area ratios were calculated for each target analyte present in the QC samples. Metabolites showing RSDs higher than 30% on peak area ratios in QC samples were excluded from further analysis (**Table S1**).

**References**

1. Sanchez-Delgado G, Alcantara JMA, Ortiz-Alvarez L, Xu H, Martinez-Tellez B, Labayen I, et al. Reliability of resting metabolic rate measurements in young adults: Impact of methods for data analysis. Clin Nutr. 2018;37:1618–24.

2. Alcantara JMA, Sanchez-Delgado G, Martinez-Tellez B, Merchan-Ramirez E, Labayen I, Ruiz JR. Congruent validity and inter-day reliability of two breath by breath metabolic carts to measure resting metabolic rate in young adults. Nutr Metab Cardiovasc Dis. 2018;28:929–36.

3. Fullmer S, Benson-Davies S, Earthman CP, Frankenfield DC, Gradwell E, Lee PSP, et al. Evidence Analysis Library Review of Best Practices for Performing Indirect Calorimetry in Healthy and Non-Critically Ill Individuals. J Acad Nutr Diet. 2015;115:1417-1446.e2.

4. Schindelin J, Arganda-Carreras I, Frise E, Kaynig V, Longair M, Pietzsch T, et al. Fiji: An open-source platform for biological-image analysis. Nat Methods. 2012;9:676–82.

5. Chen KY, Cypess AM, Laughlin MR, Haft CR, Hu HH, Bredella MA, et al. Brown Adipose Reporting Criteria in Imaging STudies (BARCIST 1.0): Recommendations for Standardized FDG-PET/CT Experiments in Humans. Cell Metab. United States; 2016;24:210–22.

6. Martinez-Tellez B, Sanchez-Delgado G, Garcia-Rivero Y, Alcantara JMA, Martinez-Avila WD, Muñoz-Hernandez M V., et al. A new personalized cooling protocol to activate brown adipose tissue in young adults. Front Physiol. 2017;8:1–10.

7. Martinez-Tellez B, Nahon KJ, Sanchez-Delgado G, Abreu-Vieira G, Llamas-Elvira JM, Van Velden FHP, et al. The impact of using BARCIST 1.0 criteria on quantification of BAT volume and activity in three independent cohorts of adults. Sci Rep. 2018;8:1–8.

8. Sanchez-delgado G, Martinez-tellez B, Olza J, Aguilera CM, Labayen I, Ortega FB, et al. Activating brown adipose tissue through exercise ( ACTIBATE ) in young adults : Rationale , design and methodology. Contemp Clin Trials. Elsevier Inc.; 2015;45:416–25.

9. Midgley AW, McNaughton LR, Polman R, Marchant D. Criteria for determination of maximal oxygen uptake: A brief critique and recommendations for future research. Sport Med. 2007;37:1019–28.

10. Henriksson H, Henriksson P, Tynelius P, Ortega FB. Muscular weakness in adolescence is associated with disability 30 years later: a population-based cohort study of 1.2 million men. Br J Sports Med. 2019;53:1221–30.

11. Bonora E, Targher G, Alberiche M, Bonadonna RC, Saggiani F, Zenere MB, et al. Homeostasis model assessment closely mirrors the glucose clamp technique in the assessment of insulin sensitivity: studies in subjects with various degrees of glucose tolerance and insulin sensitivity. Diabetes Care. 2000;23:57–63.

12. Kannan S, Mahadevan S, Ramji B, Jayapaul M, Kumaravel V. LDL-cholesterol: Friedewald calculated versus direct measurement-study from a large Indian laboratory database. Indian J Endocrinol Metab. 2014;18:502–4.

13. Serena C, Ceperuelo-Mallafré V, Keiran N, Queipo-Ortuño MI, Bernal R, Gomez-Huelgas R, et al. Elevated circulating levels of succinate in human obesity are linked to specific gut microbiota. ISME J. Springer US; 2018;12:1642–57.

14. Ceperuelo-Mallafré V, Llauradó G, Keiran N, Benaiges E, Astiarraga B, Martínez L, et al. Preoperative Circulating Succinate Levels as a Biomarker for Diabetes Remission After Bariatric Surgery. Diabetes Care. 2019;42:1956–65.

15. Expert Panel on Detection, Evaluation, and Treatment of High Blood Cholesterol in Adults E and T of HBC in A. Executive Summary of the Third Report of the National Cholesterol Education Program (NCEP) Expert Panel on Detection, Evaluation, and Treatment of High Blood Cholesterol in Adults (Adult Treatment Panel III). JAMA J Am Med Assoc. American Medical Association; 2001;285:2486–97.

16. Pr Herlemann D, Labrenz M, Jü Rgens K, Bertilsson S, Waniek JJ, Andersson AF. Transitions in bacterial communities along the 2000 km salinity gradient of the Baltic Sea. ISME J. 2011;5:1571–9.

17. Callahan BJ, Mcmurdie PJ, Rosen MJ, Han AW, Johnson AJA, Holmes SP. DADA2: High resolution sample inference from Illumina amplicon data. Nat Methods. 2016;13:581–3.

18. R Core Team. R: A Language and Environment for Statistical Computing. 2019;

19. McMurdie PJ, Holmes S. phyloseq: An R Package for Reproducible Interactive Analysis and Graphics of Microbiome Census Data. Watson M, editor. PLoS One. Public Library of Science; 2013;8:e61217.

20. Wang Q, Garrity GM, Tiedje JM, Cole JR. Naïve Bayesian Classifier for Rapid Assignment of rRNA Sequences into the New Bacterial Taxonomy. Appl Environ Microbiol. 2007;73:5261–7.

21. Cole JR, Wang Q, Fish JA, Chai B, McGarrell DM, Sun Y, et al. Ribosomal Database Project: data and tools for high throughput rRNA analysis. Nucleic Acids Res. 2014;42:D633–42.

22. Schulz C, Schütte K, Koch N, Vilchez-Vargas R, Wos-Oxley ML, Oxley APA, et al. The active bacterial assemblages of the upper GI tract in individuals with and without Helicobacter infection.

23. Lozupone CA, Knight R. Species divergence and the measurement of microbial diversity. FEMS Microbiol. Rev. NIH Public Access; 2008. p. 557–78.

24. Finotello F, Mastrorilli E, Di Camillo B. Measuring the diversity of the human microbiota with targeted next-generation sequencing. Brief Bioinform. 2018;19:679–92.

25. Leo L, Shetty S. microbiome R package. 2019.

26. Gotelli NJ, Colwell RK. Estimating species richness. Biol Divers Front Meas Assess. 2011;12:39–54.

27. Magurran AE. Measuring Biological Diversity. Oxford, United Kingdom.: Blackwell; 2004.

28. Simpson EH. Measurement of diversity. Nature. Nature Publishing Group; 1949. p. 688.

29. Camargo JA. New diversity index for assessing structural alterations in aquatic communities. Bull Environ Contam Toxicol. 1992;48:428–34.

30. Oksanen J, Blanchet FG, Friendly M, Kindt R, Legendre P, Mcglinn D, et al. “vegan”:Community Ecology Package. 2019.

31. Bray JR, Curtis JT. An Ordination of the Upland Forest Communities of Southern Wisconsin. Ecol Monogr. Wiley; 1957;27:325–49.

32. Hammer Ø, Harper DAT. PAST. Paleontological Statistics. Version 2.07. Reference manual. Blackwell Publ. 2006;351.

33. Jurado-Fasoli L, Merchan-Ramirez E, Martinez-Tellez B, Acosta FM, Sanchez-Delgado G, Amaro-Gahete FJ, et al. Association between dietary factors and brown adipose tissue volume/18F-FDG uptake in young adults. Clin Nutr [Internet]. 2020; Available from: https://linkinghub.elsevier.com/retrieve/pii/S0261561420304763

34. Di Zazzo A, Yang W, Coassin M, Micera A, Antonini M, Piccinni F, et al. Signaling lipids as diagnostic biomarkers for ocular surface cicatrizing conjunctivitis. J Mol Med. Journal of Molecular Medicine; 2020;98:751–60.

35. Van Der Kloet FM, Bobeldijk I, Verheij ER, Jellema RH. Analytical error reduction using single point calibration for accurate and precise metabolomic phenotyping. J Proteome Res. 2009;8:5132–41.

36. Fernández-Veledo S, Vendrell J. Gut microbiota-derived succinate: Friend or foe in human metabolic diseases? Rev Endocr Metab Disord. Reviews in Endocrine and Metabolic Disorders; 2019;20:439–47.
