# Supplementary material for: Elevated plasma succinate levels are linked to higher cardiovascular disease risk factors in young adults
Source: Cardiovasc Diabetol. 2021 Jul 27;20:151. doi: 10.1186/s12933-021-01333-3 (PMC8314524; doi:10.1186/s12933-021-01333-3)
Supplement: Supplementary file 2 — Additional file 2: Table S1. List of metabolites analyzed by LC-MS/MS. [file 12933_2021_1333_MOESM2_ESM.docx]

**ADDITIONAL FILE 2**

**Table S1.** List of metabolites analyzed by LC-MS/MS

| Abbreviation | IUPAC Name | ChEBI ID | RSD in QC samples |
| --- | --- | --- | --- |
| *Omega-3 oxylipins* | |  |  |
| ALA | 9Z,12Z,15Z-octadecatrienoic acid | 27432 | 9.80% |
| 9-HpOTrE | 9S-hydroperoxy-10E,12Z,15Z-octadecatrienoic acid | 165791 | NM |
| 9-HOTrE | 9S-hydroxy-10E,12Z,15Z-octadecatrienoic acid | 80447 | 7.60% |
| 12,13-DiHODE | (±)-12,13-dihydroxy-9Z,15Z-octadecadienoic acid | 88461 | 5.80% |
| EPA | 5Z,8Z,11Z,14Z,17Z-eicosapentaenoic acid | 28364 | 8.50% |
| 5-HpEPE | 5S-hydroperoxy-6E,8Z,11Z,14Z,17Z-eicosapentaenoic acid | 145815 | NM |
| 5-HEPE | (±)-5-hydroxy-6E,8Z,11Z,14Z,17Z-eicosapentaenoic acid | 72801 | 13.10% |
| 12-HpEPE | 12S-hydroperoxy-5Z,8Z,10E,14Z,17Z-eicosapentaenoic acid | 78909 | NM |
| 12-HEPE | (±)-12-hydroxy-5Z,8Z,10E,14Z,17Z-eicosapentaenoic acid | 72645 | 12.10% |
| 14,15-EpETE | (±)-14,15-epoxy-5Z,8Z,11Z,17Z-eicosatetraenoic acid | 88457 | NM |
| 14,15-DiHETE | (±)-14,15-dihydroxy-5Z,8Z,11Z,17Z-eicosatetraenoic acid | 88459 | 8.00% |
| 17,18-EpETE | (±)-17,18-epoxy-5Z,8Z,11Z,14Z-eicosatetraenoic acid | 72853 | NM |
| 17,18-DiHETE | (±)-17,18-dihydroxy-5Z,8Z,11Z,14Z-eicosatetraenoic acid | 88349 | 9.10% |
| DPA | 7Z,10Z,13Z,16Z,19Z-docosapentaenoic acid | 61204 | 14.00% |
| DHA | 4Z,7Z,10Z,13Z,16Z,19Z-docosahexaenoic acid | 28125 | 9.80% |
| 4-HDoHE | (±)-4-hydroxy-5E,7Z,10Z,13Z,16Z,19Z-docosahexaenoic acid | 72624 | 14.30% |
| 8-HDoHE | (±)-8-hydroxy-4Z,6E,10Z,13Z,16Z,19Z-docosahexaenoic acid | 72610 | 19.70% |
| 11-HDoHE | (±)-11-hydroxy-4Z,7Z,9E,13Z,16Z,19Z-docosahexaenoic acid | 72794 | 17.10% |
| 13-HDoHE | (±)-13-hydroxy-4Z,7Z,10Z,14E,16Z,19Z-docosahexaenoic acid | 72608 | 12.30% |
| 14-HDoHE | (±)-14-hydroxy-4Z,7Z,10Z,12E,16Z,19Z-docosahexaenoic acid | 72647 | 14.70% |
| 16-HDoHE | (±)-16-hydroxy-4Z,7Z,10Z,13Z,17E,19Z-docosahexaenoic acid | 72613 | 15.40% |
| 17-HDoHE | (±)-17-hydroxy-4Z,7Z,10Z,13Z,15E,19Z-docosahexaenoic acid | 72637 | 9.00% |
| 20-HDoHE | (±)-20-hydroxy-4Z,7Z,10Z,13Z,16Z,18E-docosahexaenoic acid | 72615 | 23.60% |
| 19,20-EpDPE | (±)-19(20)-epoxy-4Z,7Z,10Z,13Z,16Z-docosapentaenoic acid | 72653 | 13.50% |
| 19,20-DiHDPA | (±)-19,20-dihydroxy-4Z,7Z,10Z,13Z,16Z-docosapentaenoic acid | 72657 | 7.90% |
| *Omega-6 oxylipins* | |  |  |
| LA | 9Z,12Z-octadecadienoic acid | 17351 | 10.20% |
| 10-NO2-LA | 10-nitro,9Z,12Z-octadecadienoic acid | 34125 | 13.30% |
| 9-HPODE | (±)9-hydroperoxy-10E,12Z-octadecadienoic acid | 165782 | NM |
| 9-HODE | (±)-9-hydroxy-10E,12Z-octadecadienoic acid | 72651 | 7.60% |
| 9,12,13-TriHOME | 9S,12S,13S-trihydroxy-10E-octadecenoic acid | 34506 | 6.90% |
| 9,10,13-TriHOME | 9S,10S,13S-trihydroxy-11E-octadecenoic acid | 34499 | 15.70% |
| 13-HPODE | (±)13-hydroperoxy-9Z,11E-octadecadienoic acid | 91272 | NM |
| 13-HODE | (±)-13-hydroxy-9Z,11E-octadecadienoic acid | 72639 | 7.10% |
| 9,10-EpOME | 9,10-epoxy-12Z-octadecenoic acid | 34494 | 7.80% |
| 12,13-EpOME | (±)-12(13)-epoxy-9Z-octadecenoic acid | 38229 | 9.60% |
| 9,10-DiHOME | 9,10-dihydroxy-12Z-octadecenoic acid | 72663 | 7.30% |
| 12,13-DiHOME | 12,13-dihydroxy-9Z-octadecenoic acid | 72665 | 6.70% |
| DGLA | 8Z,11Z,14Z-eicosatrienoic acid | 53486 | 23.90% |
| 8-HETrE | 8S-hydroxy-9E,11Z,14Z-eicosatrienoic acid | 140473 | 22.80% |
| 15-HETrE | 15S-hydroxy-8Z,11Z,13E-eicosatrienoic acid | 88348 | 13.50% |
| AA | 5Z,8Z,11Z,14Z-eicosatetraenoic acid | 15843 | 13.40% |
| AdrA | 7Z,10Z,13Z,16Z-docosatetraenoic acid | 53487 | 22.10% |
| PGG2 | 9S,11R-Epidioxy-15S-hydroperoxy-5Z,13E-prostadienoic acid | 27647 | NM |
| PGH2 | 9S,11R-Epidioxy-15S-hydroxy-5Z,13E-prostadienoic acid | 15554 | NM |
| PGE2 | 9-oxo-11R,15S-dihydroxy-5Z,13E-prostadienoic acid | 15551 | 36.00% |
| PGF2alpha | 9α,11α,15S-trihydroxy-prosta-5Z,13E-dien-1-oic acid | 15553 | ND |
| TxA2 | 9S,11S-Epoxy,15S-hydroxy-thromboxa-5Z,13E-dien-1-Oic acid | 15627 | NM |
| TxB2 | 9S,11,15S-trihydroxy-thromboxa-5Z,13E-dien-1-oic acid | 28728 | 6.90% |
| 8,9-EpETrE | 8,9-epoxy-5Z,11Z,14Z-eicosatrienoic acid | 34490 | ND |
| 11,12-EpETrE | 11,12-epoxy-5Z,8Z,14Z-eicosatrienoic acid | 34130 | ND |
| 11,12-DiHETrE | 11,12-dihydroxy-5Z,8Z,14Z-eicosatrienoic acid | 63969 | 8.40% |
| 14,15-EpETrE | 14,15-epoxy-5Z,8Z,11Z-eicosatrienoic acid | 34157 | 19.50% |
| 5,6-EpETrE | (±)5,6-epoxy-8Z,11Z,14Z-eicosatrienoic acid | 34450 | NM |
| 5,6-DiHETrE | 5,6-dihydroxy-8Z,11Z,14Z-eicosatrienoic acid | 63974 | 9.00% |
| 8,9-DiHETrE | 8,9-dihydroxy-5Z,11Z,14Z-eicosatrienoic acid | 63970 | 9.90% |
| 11,12-DiHETrE | 11,12-dihydroxy-5Z,8Z,14Z-eicosatrienoic acid | 63969 | 8.40% |
| 14,15-DiHETrE | 14,15-dihydroxy-5Z,8Z,11Z-eicosatrienoic acid | 63966 | 7.10% |
| 5-HPETE | 5S-hydroperoxy-6E,8Z,11Z,14Z-eicosatetraenoic acid | 91268 | NM |
| 5-HETE | 5-hydroxy-6E,8Z,11Z,14Z-eicosatetraenoic acid | 28209 | 10.40% |
| 11-HPETE | 11R-Hydroperoxy-5Z,8Z,12E,14Z-eicosatetraenoic acid | 165279 | NM |
| 11-HETE | 11-hydroxy-5Z,8Z,12E,14Z-eicosatetraenoic acid | 72606 | 10.70% |
| 12-HPETE | 12S-hydroperoxy-5Z,8Z,10E,14Z-eicosatetraenoic acid | 15626 | NM |
| 12-HETE | 12-hydroxy-5Z,8Z,10E,14Z-eicosatetraenoic acid | 19138 | 11.40% |
| 15-HPETE | 15S-hydroperoxy-5Z,8Z,11Z,13E-eicosatetraenoic acid | 91271 | NM |
| 15-HETE | 15-hydroxy-5Z,8Z,11Z,13E-eicosatetraenoic acid | 64017 | 9.30% |
| 20-HETE | 20-hydroxy-5Z,8Z,11Z,14Z-eicosatetraenoic acid | 34306 | 9.40% |
| 1a,1b-dihomo-PGF2α | 1a,1b-dihomo-9S,11R,15S-trihydroxy-5Z,13E-prostadienoic acid | NA | 22.10% |
| 2,3-dinor-8-iso-PGF2α | 9α,11α,15S-trihydroxy-2,3-dinor-(8β)-prosta-5Z,13E-dien-1-oic acid | NA | ND |
| 2,3-dinor-11β-PGF2α | 9α,11β,15S-trihydroxy-2,3-dinor-prosta-5Z,13E-dien-1-oic acid | NA | ND |
| iPF2α-IV | (8S)-10-[(1R,2S,3S,5R)-3,5-Dihydroxy-2-pentylcyclopentyl]-8-hydroxydeca-5,9-dienoic acid | NA | ND |
| 5-iPF2α VI | (8β)-5,9α,11α-trihydroxy-prosta-6E,14Z-dien-1-oic acid | 140933 | ND |
| 8,12-iPF2α | (12α)-5,9α,11α-trihydroxy-prosta-6E,14Z-dien-1-oic acid | NA | 7.20% |
| 12-HHTrE | 12S-hydroxy-5Z,8E,10E-heptadecatrienoic acid | 63977 | 8.10% |
| 20-hydroxy-PGF2a | 9α,11α,15S,20-tetrahydroxy-prosta-5Z,13E-dien-1-oic acid | 165322 | ND |
| 20-hydroxy-PGE2 | 9-oxo-11α,15S,20-trihydroxy-prosta-5Z,13E-dien-1-oic acid | 137370 | ND |
| 8-iso-PGF2a | 9α,11α,15S-trihydroxy-(8β)-prosta-5Z,13E-dien-1-oic acid | 34509 | ND |
| 8-iso-15-R-PGF2a | 9α,11α,15R-trihydroxy-(8β)-prosta-5Z,13E-dien-1-oic acid | NA | ND |
| 11beta-PGF2a | 9α,11β,15S-trihydroxy-prosta-5Z,13E-dien-1-oic acid | 27595 | ND |
| PGF2alpha | 9S,11R,15S-trihydroxy-5Z,13E-prostadienoic acid | 15553 | ND |
| PGE3 | 9-oxo-11α,15S-dihydroxy-prosta-5Z,13E,17Z-trien-1-oic acid | 28031 | ND |
| PGD3 | 9α,15S-dihydroxy-11-oxo-prosta-5Z,13E,17Z-trien-1-oic acid | 34939 | ND |
| 8-iso-PGE2 | 9-oxo-11α,15S-dihydroxy-(8β)-prosta-5Z,13E-dien-1-oic acid | 131888 | ND |
| 11beta-PGE2 | 9-oxo-11β,15S-dihydroxy-prosta-5Z,13E-dien-1-oic acid | 89581 | ND |
| PGD2 | 9α,15S-dihydroxy-11-oxo-prosta-5Z,13E-dien-1-oic acid | 15555 | ND |
| 8-iso-13,14-dihydro-15-keto-PGF2a | 9α,11α-dihydroxy-15-oxo-(8β)-prost-5Z-en-1-oic acid | NA | ND |
| 13,14-dihydro-15-keto-PGF2a | 9α,11α-dihydroxy-15-oxo-prost-5Z-en-1-oic acid | 63976 | ND |
| 13,14-dihydro-PGF2a | 9α,11α,15S-trihydroxy-prost-5Z-en-1-oic acid | 88346 | ND |
| 13,14-dihydro-15-keto-PGE2 | 9,15-dioxo-11α-hydroxy-prost-5Z-en-1-oic acid | 15550 | ND |
| 13,14-dihydro-15-keto-PGD2 | 9α-hydroxy-11,15-dioxo-prost-5Z-en-1-oic acid | 72603 | ND |
| 1a,1b-dihomo-PGF2a | 9α,11α,15S-trihydroxy-1a,1b-dihomo-prosta-5Z,13E-dien-1-oic acid | NA | ND |
| bicyclo-PGE2 | 11-deoxy-13,14-dihydro-15-keto-11β,16. xi.-cycloprostaglandin E2 | 89568 | ND |
| 5S,6R-LipoxinA4 | 5S,6R,15S-trihydroxy-7E,9E,11Z,13E-eicosatetraenoic acid | 6498 | ND |
| 5S,6S-LipoxinA4 | 5S,6S,15S-trihydroxy-7E,9E,11Z,13E-eicosatetraenoic acid | 63990 | ND |
| 20-carboxy-LTB4 | 5S,12R-dihydroxy-6Z,8E,10E,14Z-eicosatetraene-1,20-dioic acid | 27562 | ND |
| 20-hydroxy-LTB4 | 5S,12R,20-trihydroxy-6Z,8E,10E,14Z-eicosatetraenoic acid | 15646 | ND |
| 10S,17S-DiHDoHE | 10(S),17(S)-dihydroxy-4Z,7Z,11E,13Z,15E,19Z-docosahexaenoic acid | 138653 | ND |
| 18-HEPE | (±)-18-hydroxy-5Z,8Z,11Z,14Z,16E-eicosapentaenoic acid | 72802 | ND |
| 15-HEPE | (±)-15-hydroxy-5Z,8Z,11Z,13E,17Z-eicosapentaenoic acid | 72627 | ND |
| 9-HEPE | (±)-9-hydroxy-5Z,7E,11Z,14Z,17Z-eicosapentaenoic acid | 89570 | ND |

ChEBI: Chemical Entities of Biological Interest; IUPAC, International Union of Pure and Applied Chemistry; NA: not available, ND: not detected, NM: not measured, QC: quality control, RSD: relative standard error.
