# Supplementary material for: Elevated plasma succinate levels are linked to higher cardiovascular disease risk factors in young adults
Source: Cardiovasc Diabetol. 2021 Jul 27;20:151. doi: 10.1186/s12933-021-01333-3 (PMC8314524; doi:10.1186/s12933-021-01333-3)
Supplement: Supplementary file 3 — Additional file 3: Table S2. Relative abundance (%) of species belonging to the Bacteroides and Acidaminococcus genera by tertiles of plasma succinate (n = 58). [file 12933_2021_1333_MOESM3_ESM.docx]

**ADDITIONAL FILE 3**

**Table S2.** Relative abundance (%) of species belonging to the *Bacteroides* and *Acidaminococcus* genera by tertiles of plasma succinate (n=58)

|  | **Plasma succinate tertiles** | | | | | | | | |  |
| --- | --- | --- | --- | --- | --- | --- | --- | --- | --- | --- |
|  | Low (11.6–57.3 µM) n=19 | | | Intermediate (57.8–75.4 µM) n=20 | | | High (76.3–129.8 µM) n=19 | | |  |
| *Bacteroides* genus (%) |  |  |  |  |  |  |  |  |  | P |
| *Bacteroides caccae* | 0.62 | ± | 1.26 | 0.40 | ± | 0.50 | 0.66 | ± | 1.10 | 0.729 |
| *Bacteroides cellulosilyticus* | 0.70 | ± | 1.78 | 0.15 | ± | 0.23 | 0.42 | ± | 0.59 | 0.776 |
| *Bacteroides clarus* | 0.00 | ± | 0.00 | 0.04 | ± | 0.08 | 0.03 | ± | 0.07 | 0.099 |
| *Bacteroides coprocola* | 1.25 | ± | 2.36 | 1.79 | ± | 3.21 | 0.26 | ± | 0.84 | 0.264 |
| *Bacteroides coprophilus* | 0.01 | ± | 0.03 | 0.32 | ± | 1.43 | 0.11 | ± | 0.50 | 0.462 |
| *Bacteroides dorei* | 3.33 | ± | 4.54 | 2.57 | ± | 3.79 | 4.69 | ± | 5.40 | 0.449 |
| *Bacteroides eggerthii* | 0.38 | ± | 1.42 | 0.09 | ± | 0.18 | 0.41 | ± | 0.81 | 0.730 |
| *Bacteroides faecis* | 0.32 | ± | 0.58 | 0.21 | ± | 0.41 | 0.10 | ± | 0.24 | 0.176 |
| *Bacteroides finegoldii* | 0.32 | ± | 1.06 | 0.04 | ± | 0.10 | 0.16 | ± | 0.41 | 0.796 |
| *Bacteroides fragilis* | 0.66 | ± | 1.65 | 0.15 | ± | 0.37 | 0.29 | ± | 0.70 | 0.408 |
| *Bacteroides intestinalis* | 0.02 | ± | 0.09 | 0.01 | ± | 0.02 | 0.01 | ± | 0.02 | 0.435 |
| *Bacteroides massiliensis* | 1.40 | ± | 2.67 | 1.00 | ± | 1.42 | 0.87 | ± | 1.36 | 0.853 |
| *Bacteroides nordii* | 0.00 | ± | 0.01 | 0.01 | ± | 0.02 | 0.00 | ± | 0.01 | 0.692 |
| *Bacteroides ovatus* | 0.45 | ± | 0.85 | 0.44 | ± | 1.17 | 0.15 | ± | 0.14 | 0.537 |
| *Bacteroides plebeius* | 0.54 | ± | 0.92 | 0.45 | ± | 1.63 | 0.16 | ± | 0.38 | 0.696 |
| *Bacteroides salyersiae* | 0.10 | ± | 0.38 | 0.02 | ± | 0.07 | 0.00 | ± | 0.01 | 0.635 |
| *Bacteroides sartorii* | 0.00 | ± | 0.00 | 0.02 | ± | 0.10 | 0.00 | ± | 0.00 | 0.387 |
| *Bacteroides stercoris* | 0.38 | ± | 1.01 | 1.05 | ± | 3.55 | 0.14 | ± | 0.32 | 0.741 |
| *Bacteroides thetaiotaomicron* | 0.14 | ± | 0.21 | 0.18 | ± | 0.28 | 0.50 | ± | 0.86 | 0.139 |
| *Bacteroides uniformis* | 3.37 | ± | 2.84 | 2.34 | ± | 3.01 | 3.80 | ± | 3.15 | 0.209 |
| *Bacteroides vulgatus* | 6.94 | ± | 7.06 | 2.42 | ± | 2.67 | 4.90 | ± | 5.56 | 0.186 |
| *Bacteroides xylanisolvens* | 0.44 | ± | 0.62 | 0.27 | ± | 0.41 | 0.58 | ± | 0.83 | 0.234 |
| *Acidaminococcus* genus (%) |  |  |  |  |  |  |  |  |  |  |
| *Acidaminococcus fermentans* | 0.00 | ± | 0.00 | 0.00 | ± | 0.00 | 0.33 | ± | 1.43 | 0.358 |
| *Acidaminococcus intestini* | 0.08 | ± | 0.26 | 0.92 | ± | 1.80 | 0.89 | ± | 1.54 | 0.086 |

Data are presented as mean and standard deviation (SD). P-value from the Kruskal-Wallis test, correcting for multiple comparisons FDR (P≤0.05).
