# Supplementary material for: Elevated plasma succinate levels are linked to higher cardiovascular disease risk factors in young adults
Source: Cardiovasc Diabetol. 2021 Jul 27;20:151. doi: 10.1186/s12933-021-01333-3 (PMC8314524; doi:10.1186/s12933-021-01333-3)
Supplement: Supplementary file 4 — Additional file 4: Table S3. Relative abundance (%) of succinate-producing and -consuming species previously described by Serena C. et al. by tertiles of plasma succinate (n = 58). [file 12933_2021_1333_MOESM4_ESM.docx]

**ADDITIONAL FILE 4**

**Table S3.** Relative abundance (%) of succinate-producing and -consuming species previously described by Serena C. et al. [36] by tertiles of plasma succinate (n=58)

|  | **Plasma succinate tertiles** | | | | | | | | |  |
| --- | --- | --- | --- | --- | --- | --- | --- | --- | --- | --- |
|  | Low (11.6–57.3 µM) n=19 | | | Intermediate (57.8–75.4 µM) n=20 | | | High (76.3–129.8 µM) n=19 | | |  |
| Succinate-producers (%) |  |  |  |  |  |  |  |  |  | P |
| *Bacteroides fragilis* | 0.66 | ± | 1.65 | 0.15 | ± | 0.37 | 0.29 | ± | 0.70 | 0.408 |
| *Bacteroides vulgatus* | 6.94 | ± | 7.06 | 2.42 | ± | 2.67 | 4.90 | ± | 5.56 | 0.186 |
| *Parabacteroides distasonis* | 0.36 | ± | 0.56 | 1.45 | ± | 4.48 | 0.46 | ± | 0.76 | 0.491 |
| *Paraprevotella xylaniphila* | 0.34 | ± | 1.48 | 0.08 | ± | 0.28 | 0.08 | ± | 0.33 | 0.606 |
| *Alistipes indistinctus* | 0.07 | ± | 0.13 | 0.18 | ± | 0.42 | 0.11 | ± | 0.22 | 0.467 |
| *Blautia wexlerae* | 0.31 | ± | 0.36 | 0.71 | ± | 1.85 | 0.32 | ± | 0.52 | 0.437 |
| *Faecalibacterium prausnitzii* | 2.40 | ± | 1.38 | 3.03 | ± | 2.75 | 1.93 | ± | 1.68 | 0.298 |
| *Akkermansia muciniphila* | 0.76 | ± | 1.34 | 2.28 | ± | 4.34 | 1.53 | ± | 2.49 | 0.321 |
| Succinate-consumers (%) |  |  |  |  |  |  |  |  |  |  |
| *Bacteroides thetaiotaomicron* | 0.14 | ± | 0.21 | 0.18 | ± | 0.28 | 0.50 | ± | 0.86 | 0.139 |
| *Phascolarctobacterium faecium* | 1.39 | ± | 2.94 | 1.85 | ± | 3.82 | 2.54 | ± | 5.10 | 0.609 |
| *Phascolarctobacterium succinatutens* | 0.47 | ± | 1.42 | 0.79 | ± | 1.53 | 0.11 | ± | 0.42 | 0.712 |
| *Ruminococcus bromii* | 0.17 | ± | 0.41 | 0.42 | ± | 1.03 | 0.42 | ± | 1.34 | 0.738 |
| *Dialister propionicifaciens* | 0.03 | ± | 0.11 | 0.00 | ± | 0.01 | 0.00 | ± | 0.00 | 0.645 |
| *Dialister succinatiphilus* | 0.00 | ± | 0.00 | 0.00 | ± | 0.01 | 0.65 | ± | 2.00 | 0.354 |

Data are presented as mean and standard deviation (SD). P-value from the Kruskal-Wallis test, correcting for multiple comparisons FDR (P≤0.05).
