# Supplementary material for: Elevated plasma succinate levels are linked to higher cardiovascular disease risk factors in young adults
Source: Cardiovasc Diabetol. 2021 Jul 27;20:151. doi: 10.1186/s12933-021-01333-3 (PMC8314524; doi:10.1186/s12933-021-01333-3)
Supplement: Supplementary file 5 — Additional file 5: Table S4. Characteristics of metabolically healthy overweight/obese (MHOO, n = 27) and metabolically unhealthy overweight/obese (MUOO, n = 16) individuals. [file 12933_2021_1333_MOESM5_ESM.docx]

**ADDITIONAL FILE 5**

**Table S4.** Characteristics of metabolically healthy overweight/obese (MHOO, n=27) and metabolically unhealthy overweight/obese (MUOO, n=16) individuals

|  | MHOO | | | MUOO | | |  | P |
| --- | --- | --- | --- | --- | --- | --- | --- | --- |
| Sex (n,%) |  |  |  |  |  |  | | **0.013** |
| Men | 8 (29.6) | | | 11 (68.8) | | | |  |
| Women | 19 (70.4) | | | 5 (31.2) | | | |  |
| Age (years) | 21.9 | ± | 2.4 | 22.7 | ± | 2.5 | | 0.663 |
| BMI (kg/m^2^) | 28.2 | ± | 2.4 | 30.3 | ± | 3.1 | | 0.050 |
| LMI (kg/m^2^) | 15.7 | ± | 2 | 17.3 | ± | 1.9 | | 0.376 |
| FMI (kg/m^2^) | 11.1 | ± | 2.1 | 11.4 | ± | 2.2 | | **0.023** |
| Body fat (%) | 40.1 | ± | 6.6 | 38.5 | ± | 5.3 | | 0.071 |
| VAT (g) | 459 | ± | 147 | 570.6 | ± | 127 | | **0.036** |
| Glucose (mg/dL) | 86.9 | ± | 6.6 | 93.6 | ± | 6.9 | | **0.004** |
| Insulin (µUl/mL) | 8.7 | ± | 3.9 | 13.8 | ± | 7.2 | | **0.001** |
| HOMA index | 1.9 | ± | 1 | 3.3 | ± | 1.9 | | **0.001** |
| Total cholesterol (mg/dL) | 152.1 | ± | 21.7 | 175.6 | ± | 38 | | **0.035** |
| HDL-C (mg/dL) | 50.6 | ± | 9.3 | 42.5 | ± | 9.3 | | **0.035** |
| LDL-C (mg/dL) | 88 | ± | 16.9 | 107.6 | ± | 36 | | 0.161 |
| Triglycerides (mg/dL) | 67.6 | ± | 23.7 | 148.6 | ± | 86 | | **<0.001** |
| C-reactive protein (mg/L) | 2.7 | ± | 2.3 | 4.7 | ± | 6.3 | | 0.203 |
| SBP (mmHg) | 117.5 | ± | 7.5 | 129.3 | ± | 10 | | **0.003** |
| DBP (mmHg) | 71.4 | ± | 6.01 | 77.8 | ± | 7.3 | | **0.016** |
| Metabolic syndrome ATPIII (n,%) | 0 (0) | | | 5 (31) | | | | **0.003** |
| Cardiorespiratory fitness (mL/kg/min) | 38.3 | ± | 8.3 | 37.7 | ± | 6.4 | | **0.048** |

Data are presented as mean and standard deviation (SD), unless stated otherwise. P-value from one-way analysis of variance, which was conducted with log10 transformed data and sex adjusted, or from chi-square for categorical variables. ATP III: National Cholesterol Education Program Adult Treatment Panel III. BMI: body mass index; DBP: diastolic blood pressure; FMI: fat mass index; HDL-C: High-density lipoprotein cholesterol; HOMA: homeostatic model assessment; LDL-C: Low-density lipoprotein cholesterol; LMI: lean mass index; MHOO: metabolically healthy overweight-obese; MUOO: metabolically unhealthy overweight-obese; SBP: systolic blood pressure; VAT: visceral adipose tissue.
