# Supplementary material for: Elevated plasma succinate levels are linked to higher cardiovascular disease risk factors in young adults
Source: Cardiovasc Diabetol. 2021 Jul 27;20:151. doi: 10.1186/s12933-021-01333-3 (PMC8314524; doi:10.1186/s12933-021-01333-3)
Supplement: Supplementary file 6 — Additional file 6: Fig S1. Waterfall plot showing plasma succinate levels per individual (n=100). Each bar represents a single individual. [file 12933_2021_1333_MOESM6_ESM.docx]

**ADDITIONAL FILE 6**

**
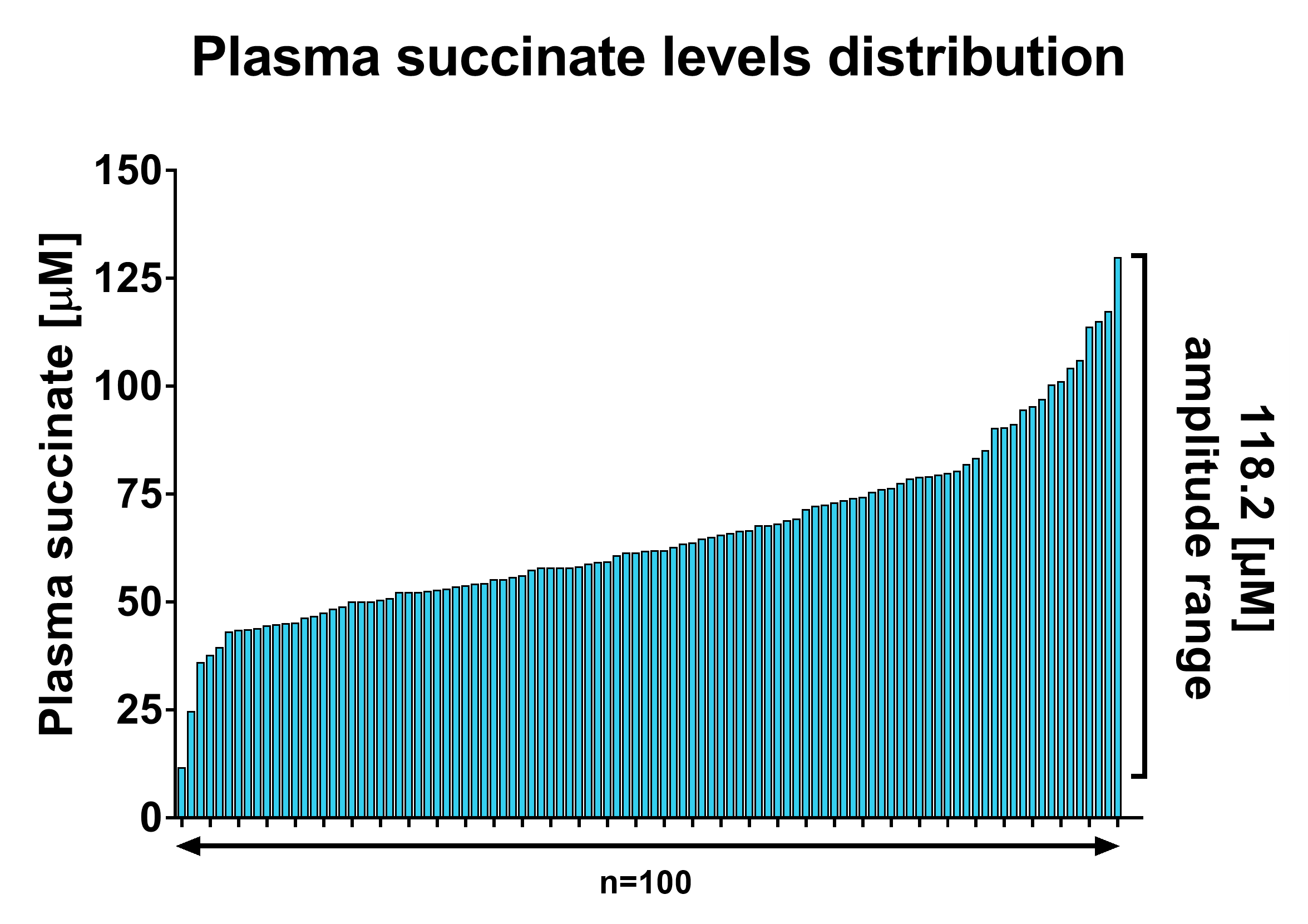
**

**Fig S1. Waterfall plot showing plasma succinate levels per individual (n=100).** Each bar represents a single individual.
