# Supplementary material for: Elevated plasma succinate levels are linked to higher cardiovascular disease risk factors in young adults
Source: Cardiovasc Diabetol. 2021 Jul 27;20:151. doi: 10.1186/s12933-021-01333-3 (PMC8314524; doi:10.1186/s12933-021-01333-3)
Supplement: Supplementary file 7 — Additional file 7: Fig. S2. Differences at family (A) and genus (B) levels by tertiles of plasma succinate levels (n = 58). [file 12933_2021_1333_MOESM7_ESM.docx]

**ADDITIONAL FILE 7**


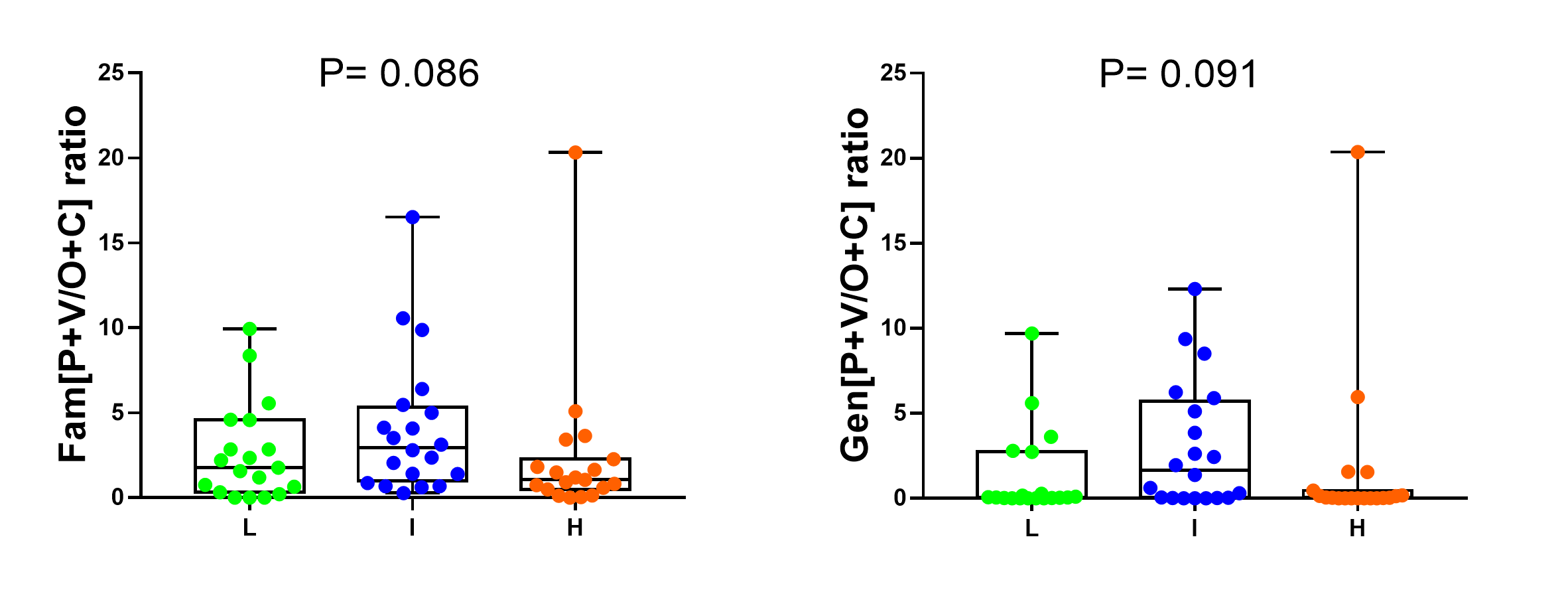


**Fig. S2. Differences at family (A) and genus (B) levels by tertiles of plasma succinate levels (n=58):** families (*Prevotellaceae* plus *Veillonellaceae/Odoribacteriaceae* plus *Clostridaceae*) [fam(P +V/O + C)] ratio; genera (*Prevotellaceae spp.* plus *Veillonellaceae spp./Odoribacteriaceae spp.* plus *Clostridaceae spp.*) [gen(P +V/O + C)] ratio. P-value from the Kruskal-Wallis test, correcting for multiple comparisons FDR (P<0.05).
