# Supplementary material for: Elevated plasma succinate levels are linked to higher cardiovascular disease risk factors in young adults
Source: Cardiovasc Diabetol. 2021 Jul 27;20:151. doi: 10.1186/s12933-021-01333-3 (PMC8314524; doi:10.1186/s12933-021-01333-3)
Supplement: Supplementary file 8 — Additional file 8: Fig. S3. Interaction network pathway analysis of circulating omega-3 and omega-6 oxylipins (n = 98) [file 12933_2021_1333_MOESM8_ESM.docx]

**ADDITIONAL FILE 8**


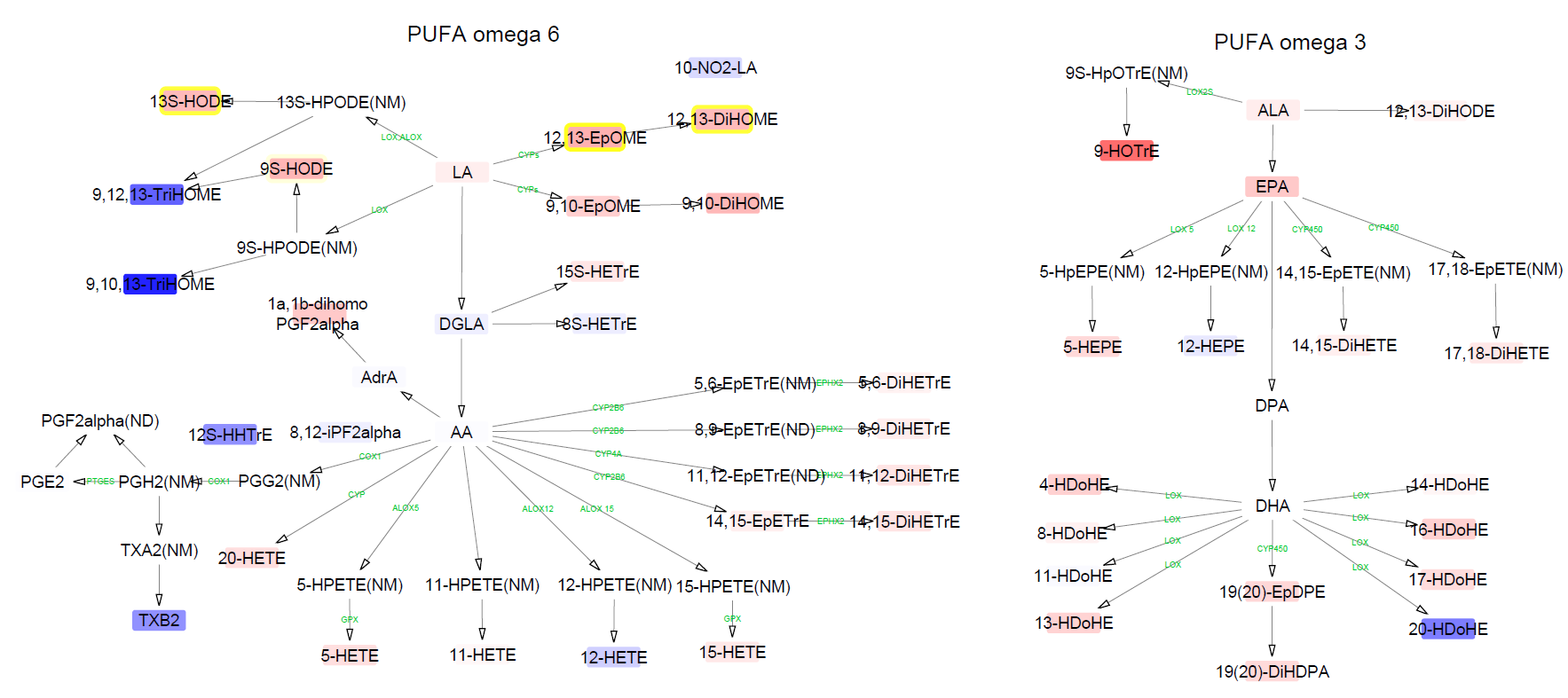


**High vs. Medium**

**A**

**Omega-6 oxylipins**

**Omega-3 oxylipins**


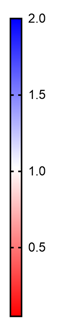


**Fold change**


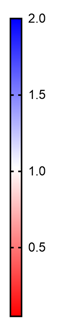


**B**

**Medium vs. Low**

**Omega-6 oxylipins**

**Omega-3 oxylipins**


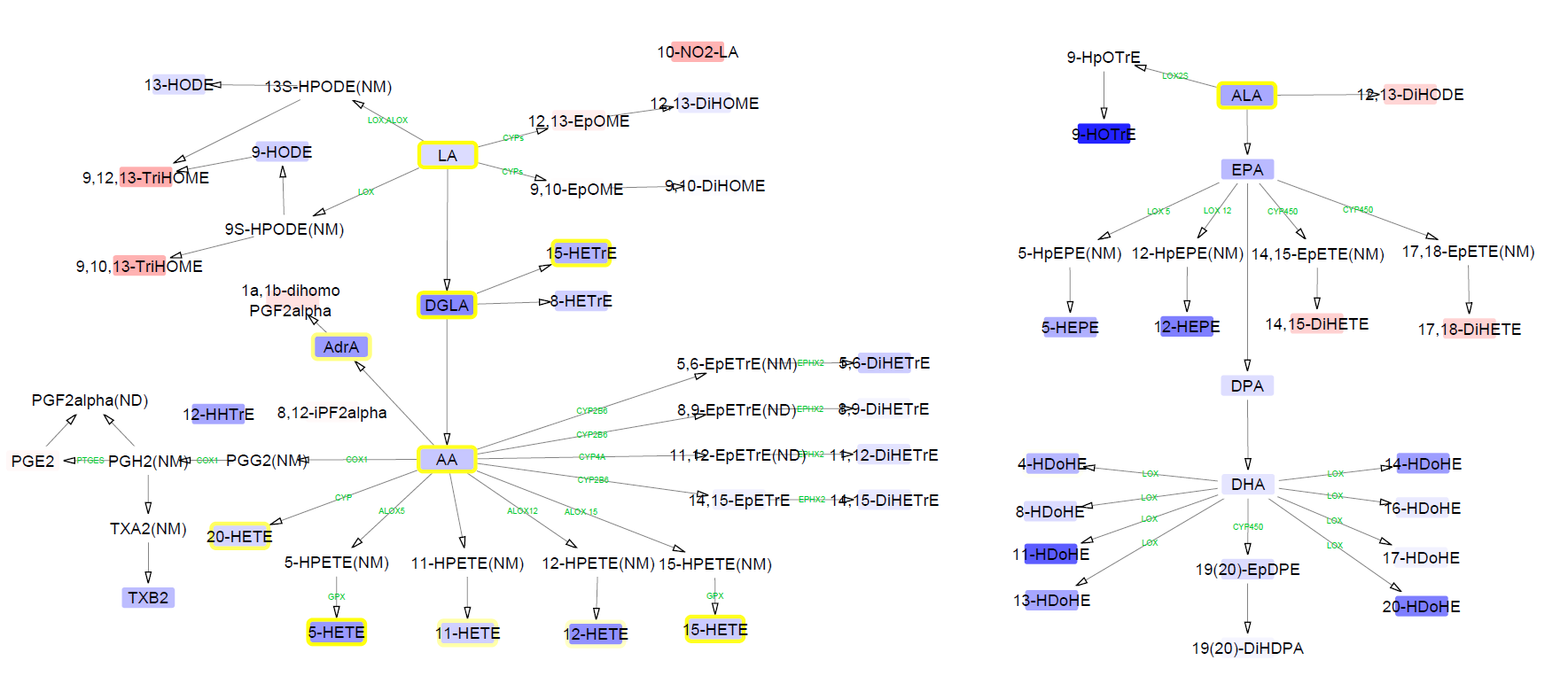


**Fold change**

**Fig. S3. Interaction network pathway analysis of** **circulating omega-3 and omega-6 oxylipins (n=98).** The networks depict the differences of each lipid mediator between high *versus* medium tertiles of plasma succinate levels (A) and medium *versus* low tertiles of plasma succinate levels. Compounds that were not detected (ND) or not measured (NM) with the LC-MS/MS method are shown without boxes. Blue boxes with yellow borderlines indicate that this lipid mediator was higher in high *versus* the low succinate group, whereas red boxes with yellow borderlines indicate that this lipid mediator was lower in the comparison. Boxes without yellow borderlines indicate that this lipid mediator did not significantly change in the comparison. Fold-change values are depicted according to the legend bar on the right side. Intermediate enzymes are represented in green and they were not measured. Comparisons are performed with independent t-test analysis (log10 transformed values) and P<0.05.
